# Eco-friendly methodology for removing and recovering rare earth elements from saline industrial wastewater

**DOI:** 10.1007/s11356-023-29088-2

**Published:** 2023-08-14

**Authors:** Thainara Viana, Nicole Ferreira, Daniela S. Tavares, Azadeh Abdolvaseei, Eduarda Pereira, Bruno Henriques

**Affiliations:** 1 LAQV-REQUIMTE - Associated Laboratory for Green Chemistry & Department of Chemistry, University of Aveiro, 3810-193 Aveiro, Portugal; 2 Central Laboratory of Analysis, University of Aveiro, 3810-193 Aveiro, Portugal

**Keywords:** Critical raw materials, Rare earth elements, Biosorption, Response surface methodology, Seaweed

## Abstract

In this study, response surface methodology (RSM) was applied with a Box–Behnken design to optimize the biosorption (removal and bioconcentration) of rare earth elements (REEs) (Y, La, Ce Eu, Gd, Tb) by living *Ulva* sp. from diluted industrial wastewaters (also containing Pt and the classic contaminants Hg, Pb, Zn, Cu, Co, and Cd). Element concentration (A: 10–190 μg/L), wastewater salinity (B: 15–35), and *Ulva* sp. dosage (C: 1.0–5.0 g/L) were the operating parameters chosen for optimization. Analysis of the Box–Behnken central point confirmed the reproducibility of the methodology and *p-*values below 0.0001 validated the developed mathematical models. The largest inter-element differences were observed at 24 h, with most REEs, Cu, Pb and Hg showing removals ≥ 50 %. The factor with the greatest impact (positive) on element removal was the initial seaweed dosage (ANOVA, *p *< 0.05). The optimal conditions for REEs removal were an initial REEs concentration of 10 μg/L, at a wastewater salinity of 15, and an *Ulva* sp. dosage of 5.0 g/L, attaining removals up to 88 % in 24 h. Extending the time to 96 h allowed seaweed dosage to be reduced to 4.2 g/L while achieving removals ≥ 90 %. The high concentrations in REE-enriched biomass (∑REEs of 3222 μg/g), which are up to 3000 times higher than those originally found in water and exceed those in common ores, support their use as an alternative source of these critical raw materials.

**Supplementary Information:**

The online version contains supplementary material available at 10.1007/s11356-023-29088-2.

## Introduction

Since China’s position in the global rare earth elements (REEs) market changed in 2009, anxiety befell among manufacturers of high-tech equipment. The unique properties of REEs led to ever-increasing demand and to severe constraints on its supply—critical raw materials (European Commission 2023). The recovery of REEs from waste electric and electronic equipment (e-waste) such as lamp/TV phosphors and batteries, or from wastewater can be an additional source of REEs and help reduce the serious environmental impacts associated with REEs extraction and improper e-waste management (Deng et al. 2022).

With the ban on incandescent lamps, fluorescent lamps (FLs) and light-emitting diodes (LED) greatly expanded (Machacek et al. 2015). Both use REEs phosphors to produce visible light (red, blue, and green) (Jiang et al. 2022), although FLs contain higher concentrations of REEs compared to LED (0.005 vs. 1.2 g/unit) (Machacek et al. 2015; Rebello et al. 2020) and are classified as hazardous waste due their Hg content. Millions of FLs are sold and discarded each year, making them an interesting holdover from a REEs recovery perspective (Zhang et al. 2017). Spent NiMH batteries are another potential source of REEs and other critical elements such as Co, Ni, and Mn, which poses an environmental hazard if not properly managed (Rasoulnia et al. 2021). Nevertheless, despite the high value of the recoverable materials in e-waste, the recycling rates have not kept pace with e-waste generation rates (1 – 12 %) (Balaram 2019).

Several methods have been described to recover REEs from e-waste and wastewater, including ion exchange (Felipe et al. 2021), solvent extraction (Xie et al. 2014), chemical precipitation (Silva et al. 2019), ionic imprinted polymers (Hu et al. 2018), among others. Nevertheless, all share disadvantages such as high operational costs, high reagent/energy consumption, production of secondary metabolites (Pereao et al. 2018), and low efficiency at relatively low REEs concentrations.

Biosorption has been highlighted as a greener, and cheaper tool to remove elements from aqueous solutions (Giese 2020). A highly efficient, economical, and environmentally friendly solution for water remediation, with low nutrient requirements, lower volume of sludge to be disposed of, and the potential for element recovery are important advantages pointed to biosorption. Available research reports the use of a variety of biosorbents such as crab shell, orange peel (Liu et al. 2021), animal (fish scales) and plant origin (neem sawdust) (Das et al. 2014), micro- and macroalgae, the latter also known as seaweed when they are marine (Kucuker et al. 2017; Viana et al. 2021). However, most studies are still conducted under ideal conditions, i.e., in distilled water, focusing on one or a few elements in unrealistically high concentrations, and also not considering the effect of ionic strength, although high salt concentrations are common in industrial effluents and decrease biosorption performance in general (Torres 2020).

In general, the influence of the operating conditions is assessed individually, but to understand the complexity of the biosorption process, it is essential to know the interactive effects of the operating variables. Response surface methodology (RSM) allows to obtain of a set of relevant information with a small set of trials (Bowden et al. 2019). The purpose of this methodology is to set up a series of experiments (design) for adequate predictions of a response and to optimize conditions to reach the highest efficiency (Whitford et al. 2018).

In the present study, the effects of three factors, initial element concentration, wastewater salinity and biosorbent dosage, on the removal and preconcentration of six rare earth elements (Y, Eu, La, Ce, Tb, and Gd) by living *Ulva* sp. were studied using the Box–Behnken design, which provided mathematical models showing the influence of each variable and their interactions. To bridge the gap between laboratory biosorption studies and real industrial effluents, a complex mixture of contaminants simulating industrial effluents (Y, Eu, La, Ce, Tb, Gd, Hg, Pb, Zn, Cu, Co, Cd, Pt) was examined.

## Materials and methods

### Seaweed collection

*Ulva* sp. (Chlorophyta (green seaweed)) is a common species that has already shown great potential to remove some potentially toxic elements (PTEs—i.e., Hg, Pb, Cd) and REEs from less complex environments (Costa et al. 2020b; Pinto et al. 2020). Seaweed was collected at low tide at Mira channel, Ria de Aveiro (Portugal, 40°38′39″N, 8°44′43″W) and was transported to the laboratory in isothermal plastic bags containing local water. After rinsing with filtered seawater to remove debris and epibionts, the living seaweed was maintained in aerated aquaria with artificial seawater of the desired salinity, under natural light (approximately 12 h light:12 h dark), and at a room temperature of 20 ± 2 °C until their use (Henriques et al. 2019).

### Reagents

All glass material was previously immersed in 25 % (v/v) nitric acid (Merck, Suprapur®, 65 % (m/v)) for at least 24 h and then rinsed with ultrapure water (18 MΩ/cm) for later use. All chemical reagents used in this work were obtained from commercial suppliers and are classified as analytical-grade reagents. Standard solutions for rare earth elements (1000 mg/L, in HNO_3_ 1.4 – 7 %) were provided by certified suppliers (Inorganic Ventures™, Alfa Aesar, PlasmaCAL and Sigma-Aldrich). The platinum standard solution (994 ± 4 mg/L, in HCl 10 % v/v) was provided by Inorganic Ventures™. Standard solutions for PTEs were supplied by Merck (1000 mg/L, in HNO_3_ 0.5 – 3 %). The desired salinity was achieved by diluting real seawater with ultrapure water. The salinity of the water was measured using a WTW 720 series multiparameter meter.

### Experimental design

The present work evaluates a complex mixture mimicking the complexity of industrial effluents and e-waste contaminated waters consisting of 13 elements (Y, Eu, La, Ce, Tb, Gd, Hg, Pb, Zn, Cu, Co, Cd, Pt) (Rajesh et al. 2022). A RSM with a Box–Behnken design (BBD) was used to study and optimize the process of water decontamination and REEs bioconcentration by *Ulva* sp*.*, avoiding time- and resource-consuming single-factor analysis (Bowden et al. 2019). The experimental conditions followed a 3-factor 3-level design with equidistant values (− 1; 0; + 1). A second-order polynomial equation was used in the modelling since BBD describes linear, quadratic and interaction effects (Witek-Krowiak et al. 2014). Element concentration in solution (A: 10, 100 and 190 μg/L), salinity (B: 15, 25 and 35), and initial seaweed dosage (C: 1, 3, and 5 g/L—fresh weight (FW)) were the factors evaluated. The conditions of the 15 experiments are detailed in the Supplementary Material—Table S1. Central point conditions (100 μg/L, salinity 25, and 3 g/L FW) were run in triplicate for the BBD evaluation.

Seawater spiked with the elements was added to 1 L Schott Duran® flasks for pre-equilibration 24 h prior to assays. NaOH (1 mol/L) was used to adjust the pH of the solutions to 7.8 – 8.0. Assays were conducted under natural light for 144 h. Solution samples with a volume of 5 – 10 mL were withdrawn immediately before the addition of the seaweed (time 0 h), and at pre-determined periods (24, 48, 96, and 144 h), and stored in previously acidified (HNO_3_ 65 % v/v) polystyrene tubes to ensure a pH < 2.

Control solutions (without seaweed) were run under the boundary conditions of salinity and initial concentration of elements (10 μg/L at salinity 15; 10 μg/L at salinity 35; 190 μg/L at salinity 15; and 190 μg/L at salinity 35) to assess possible contamination, losses, or co-precipitation of elements. All assays were performed under BBD conditions along with controls to ensure the quality control. Solution samples were stored at 4 °C and seaweed samples were stored at − 80 °C for analysis.

### Element quantification

#### Seawater

The concentration of elements in water (except for Hg—supplementary material) was measured by inductively coupled plasma mass spectrometry (ICP-MS) on a Thermo Scientific X Series instrument equipped with a Burgener nebuliser. Calibration curves were established with 5 standards (concentration between 0.1 and 10 μg/L) prepared by diluting the commercially available certified standard solutions in 2 % HNO_3_. Only correlation coefficients above 0.999 were considered. The limit of quantification was 0.1 μg/L with a coefficient of variation between replicates of no more than 5 %. To avoid matrix interferences due to the high salinity, all water samples were diluted 20-fold in 2 % HNO_3_ before analysis.

#### Seaweed

To quantify elements concentration by ICP-MS in seaweed biomass (except for Hg—supplementary material), before and after exposure assays, samples were previously lyophilized and subjected to acid digestion: 200 mg of the sample was first solubilized with 2 mL of HNO_3_ in Teflon vials (HP-1500), for a 19 min at 190 °C in a CEM Mars 5 microwave. After cooling down, 250 μL of H_2_O_2_ was added and allowed to react for 20 min before another 19 min cycle at 190 °C. The digested solutions were collected into 25 mL polyethylene vials and the volume was made up with ultrapure water.

In addition to sample replicates, blank samples (reaction vials without sample following the same digestion process) and certified reference material (SRM 1547 - peach leaves) were evaluated in parallel with seaweed samples. The recovery percentage varied between 99 and 108 %. The blank values were always below the limit of quantification.

### Data analysis

The efficiency of removing elements (R, %) was calculated from their initial concentration (C_0_, μg/L) and the concentration at time *t* (C_t_, μg/L) in the solution mimicking the lamp industry wastewater (Eq. 1):

1 $$R=\frac{\left({C}_0-{C}_t\right)}{C_0}\times 100$$

Considering that all elements were removed from the solution by the seaweed, the expected concentration in the seaweed biomass, after a time *t* of exposure to the solution (*q*_*t*_, μg/g) was calculated by mass balance (Eq. 2) (Henriques et al. 2015):

2 $${q}_t=\frac{\left({C}_0-{C}_f\right)}{m}\times V$$

where *C*_*f*_ is the final concentration in the solution, *m* (g) is the mass of seaweed in dry weight, and *V* (L) is the volume of the solution.

The bioconcentration factor (BCF), defined as the ratio between the concentration in the seaweed (*q*_real_, μg/g) and the initial concentration in the solution (*C*_0_, μg/L) was calculated using Eq. 3:

3 $$BCF=\frac{q_{real}\times 1000}{C_0}$$

Data analysis was performed using Design-Expert software (version 13.0.1, Stat-Ease Inc., Minneapolis, USA). ANOVA was used to assess the significance of the factors and the interactions between them were tested using Fisher’s test and its associated probability *p*(*F*). The coefficient of determination, *R*^2^, and the adjusted coefficient of determination, *R*^2^_Adj_, were used to check the goodness of the fits.

## Results and discussion

### Quality control and influence of time on removal

**Fig. 1:**
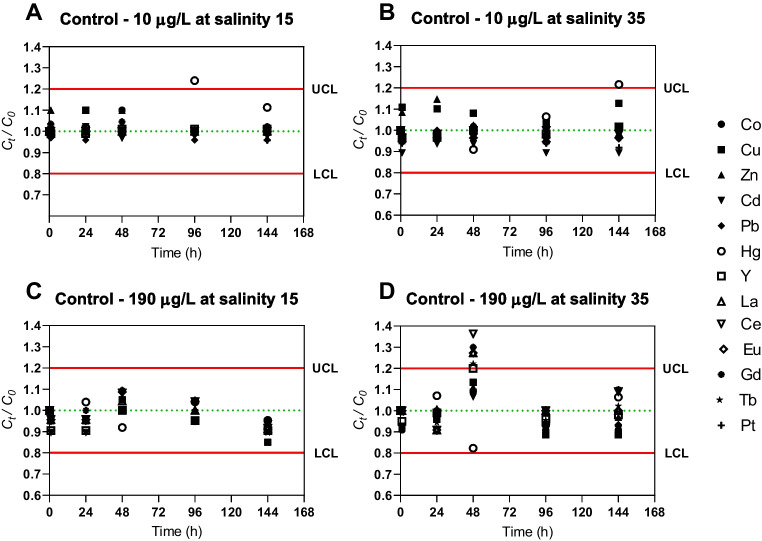
Control charts for the elements normalized concentration in control experiments over the exposure time (UCL, upper control limit; LCL, lower control limit)

Although control experiments are not preconized by BBD, four control assays were used in the present work to ensure the quality of the results (Fig. 1). A maximum acceptable variation over time of 20 % was stipulated (upper and lower limits in Fig. 1). All *C*_*t*_*/C*_*0*_ values were within the defined intervals with no relevant changes over time, indicating that losses (vessel adsorption, co-precipitation, or volatilization in the case of Hg), or contamination were negligible. Exceptions are two occasional cases for Hg (Fig. 1A and B) and a punctual case for REEs (Fig. 1D) which may attributed to uncertainties related to equipment quantification or contamination during sample collection.

**Fig. 2:**
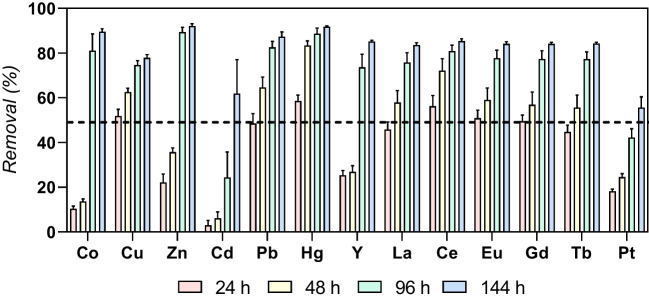
Removal (%) of the 13 elements from the mixture mimicking the elemental composition of industrial effluent, for the condition corresponding to the BBD central point (mean value ± standard deviation; *n* = 3). The black line indicates 50 % of removal

The BBD implies that central point replicates, where all the factors are in their central values, are done to assess whether the data is within the normal dispersion and repeatability is ensured. Except for Cd (for all contact times), global between-replicate coefficients of variation (CV) were ≤ 10 % for all elements at all contact times, validating the experimental design (Fig. 2).

The contact time is known to influence the efficiency of the sorption process, so its assessment is paramount to introduce the process into a real company scenario (Azubuike et al. 2016). Shorter contact times are beneficial for industries as they reduce the overall costs associated with the process.

To assess difference among contact times regarding both removal and bioconcentration (*p*-value < 0.05), a non-parametric statistical test was applied (Friedman’s test with Dunn’s multiple comparison test) (Ali and Bhaskar 2016). The results are summarized in Table S2 of the Supplementary Material. Time extensions from 24 to 48 h, 48 to 96 h, and 96 to 144 h were not significant for any of the responses evaluated. In contrast, increasing the time from 24 h to either 96 h or 144 h proved to be significant for both responses. The extension from 48 to 144 h was also significant. Based on Table S2, no significant benefits are seen in running the process for times longer than 96 h, which can be considered the most appropriate exposure time.

### Box–Behnken design data modelling

The removal efficiency (%) and the corresponding bioconcentration (μg/g) of Y, La, Ce, Eu, Gd, and Tb in *Ulva* sp. for the 15 conditions studied and times analyzed are summarized in Table 1. In terms of removal (%), the highest values for all REEs (> 94 %) were achieved in trials 3 and 9 (A: 10 μg/L; B: 25; C: 5.0 g/L and A: 10; B: 15; C: 3.0), after144 h. Cerium was the REEs with the fastest kinetic, similarly to Hg, with removals of 80 and 75 % for trials 3 and 9, respectively, after 24 h. In contrast, the lowest removals observed after 144 h exposure were 39, 71, 80, 69, 67, and 61 % for Y, La, Ce, Eu, Gd and Tb, respectively, in trial 6 (A: 100 μg/L; B: 35; C: 1.0 g/L). Costa et al. (Costa et al. 2020a) studied the removal of REEs (Y, La, Ce, Pr, Nd, Eu, Gd, Tb, Dy) from a solution in the presence and absence of some PTEs (Cr, Ni, Cu, Cd, Hg, Pb), in equimolar concentration (1 μmol/L), and observed that the presence of PTEs altered the kinetic profile, leading to an improvement in REEs removal.

**Table 1:** Removal (%) and bioconcentration (μg/g) of rare earth elements by *Ulva* sp. from the mixture mimicking the elemental composition of an industrial effluent over the exposure time

		Removal (%)						Bioconcentration (μg/g)					
Trial	Time (h)	Y	La	Ce	Eu	Gd	Tb	Y	La	Ce	Eu	Gd	Tb
1	24	26	37	48	38	38	35	12	23	26	23	22	15
	48	32	45	69	47	43	44	15	28	37	28	25	19
	96	45	60	87	65	60	63	21	37	48	40	35	27
	144	55	68	88	73	72	72	26	43	48	45	43	31
2	24	20	29	35	36	38	34	201	322	393	363	383	302
	48	32	43	49	48	49	47	322	473	544	484	494	413
	96	50	60	62	60	61	61	504	665	685	604	614	534
	144	70	76	76	76	77	77	705	846	846	766	776	675
3	24	49	69	85	75	73	70	6	9	12	10	9	8
	48	58	78	95	81	76	77	7	10	13	11	10	8
	96	-	90	96	95	94	94	-	12	13	12	12	10
	144	-	95	97	96	96	96	-	13	13	13	12	10
4	24	53	58	64	70	72	68	129	154	172	172	176	143
	48	53	68	78	73	74	70	129	181	207	178	181	147
	96	78	80	83	80	82	82	192	214	221	196	201	172
	144	88	87	88	86	87	87	216	232	234	212	214	183
5	24	29	33	40	41	38	38	165	201	267	252	216	190
	48	36	43	53	53	50	49	206	267	355	324	283	247
	96	62	68	72	71	70	70	350	417	484	437	396	355
	144	76	78	80	80	79	80	432	478	535	494	448	401
6	24	12	20	27	26	22	18	61	111	152	142	111	81
	48	14	33	45	33	29	25	71	182	253	182	152	111
	96	22	55	71	54	49	43	111	304	405	294	253	192
	144	39	71	80	69	67	61	203	395	456	375	344	273
7	24	61	68	73	75	76	73	70	86	101	95	96	77
	48	59	75	84	82	82	78	68	95	116	103	103	83
	96	86	85	88	88	89	88	99	108	122	111	112	93
	144	92	90	91	91	92	92	105	114	125	115	116	97
8	24	29	53	66	63	60	54	33	65	85	76	69	54
	48	24	56	80	57	55	48	26	69	102	69	63	48
	96	61	77	87	79	77	76	67	95	112	95	89	76
	144	91	88	91	90	90	90	101	109	116	109	104	90
9	24	49	65	75	70	68	65	9	14	16	15	13	11
	48	62	77	93	81	79	78	12	17	20	18	16	14
	96	91	90	96	94	94	94	18	19	21	20	19	16
	144	-	95	97	97	96	97	-	21	21	21	19	17
10	24	23	45	65	48	44	38	4	9	14	9	9	5
	48	27	50	87	52	49	46	4	10	18	10	10	6
	96	75	79	94	83	71	84	12	16	20	16	14	11
	144	-	90	94	93	93	94	-	19	20	18	19	13
11	24	50	50	52	54	55	55	206	225	244	244	225	206
	48	66	69	72	73	72	73	274	309	336	328	298	274
	96	86	86	86	87	87	88	356	386	405	392	358	328
	144	92	91	92	92	91	92	381	411	430	413	377	345
12	24	23	41	49	48	46	43	82	145	174	170	156	128
	48	34	58	66	60	58	55	121	206	234	209	195	163
	96	76	78	78	79	79	80	266	277	277	277	266	238
	144	80	78	78	79	79	80	280	277	277	277	266	238
13	24	27	49	58	52	51	47	47	95	117	98	91	73
	48	42	60	73	62	61	58	73	117	146	117	109	91
	96	79	77	80	79	80	79	138	149	160	149	142	123
	144	86	84	85	84	84	84	149	162	171	158	149	131
14	24	27	49	61	54	51	47	48	96	122	103	92	74
	48	44	64	76	65	63	63	78	126	151	126	114	100
	96	81	81	83	81	80	81	143	159	165	155	145	128
	144	85	84	86	84	84	84	151	164	171	161	152	133
15	24	27	46	59	55	52	47	50	96	127	112	100	77
	48	35	59	79	62	60	56	66	123	170	127	116	92
	96	76	80	84	81	81	80	143	166	182	166	156	133
	144	85	85	87	86	85	85	161	177	188	174	165	140

The highest bioconcentration (μg/g) in *Ulva* sp. was achieved in trial 2 (A: 190 μg/L; B: 25; C: 1.0 g/L), reaching values > 650 μg/g after 144 h of exposure. Such values reveal the high capability of *Ulva* sp. to preconcentrate REEs from complex effluents that enter the ecosystem. The bioconcentration factor (BCF, DW), determined as the ratio between the REEs concentration in *Ulva* sp. at 144 h and initially in solution, varied within the broad interval of 900 (Tb) and 5350 (Ce) (Supplementary Material, Table S3), with trial 5 achieving higher BCF values (lowest value of 4010 for Tb). The capability of *Ulva* sp. to concentrate REEs was previously assessed in mono (Ferreira et al. 2020) and multi-element scenarios (Costa et al. 2020a). The higher value attained in this study is 3.1 and 1.4 times higher than the reported by Ferreira et al. and Costa et al., respectively.

Thorough multiple regression analysis, mathematical correlations between factors and removal or bioconcentration were established. The goodness of fit of the models developed can be seen in the Supplementary Material (Table S4 to Table S9). ANOVA confirmed that the models are significant and suitable to predict REEs removal (Tables S4 – S9). For removal (%), the linear and quadratic terms B, C, A^2^, and C^2^ were significant for most of the quadratic models. Based on the F-value, the main effects of the independent variables followed the order of seaweed dosage, salinity, and initial concentration. Non-significant terms, such as A, AB, AC, BC, and B^2^, had limited impact on the response and were excluded from the study to improve the model (reduced model).

Since *Ulva* sp. is a euryhaline seaweed, found in brackish water conditions and estuarine substrates (Pereira 2015), it is tolerant to different salinities, being perfectly adapted to different habitat conditions, resulting in extreme resilience. Thus, the significance that the RSM attaches to salinity may be related to the different speciation of the elements in saline water rather than to the adaptability of *Ulva* sp.. This seaweed can also grow in sewage-contaminated areas, and is an opportunistic species that can form massive blooms that reflect fluctuations in environmental quality faster than slow-growing seaweed like *Fucus* (Christiansen 2018; Coelho et al. 2005). This may justify why the initial concentration of the element is not an impactful factor according to the RSM.

For bioconcentration (μg/g), the terms A, B, C, AC, and C^2^ were significant for most of the quadratic models. Based on the *F*-value, the main effects of the independent variables followed the order of initial concentration, seaweed dosage, and salinity. Non-significant terms, such as AB, BC, A^2^, and B^2^, had a limited impact on response and were excluded from the study to improve the model.

The polynomial equations developed considering only the significant factors for the responses considered (reduced models, uncoded variable values) are shown in Table 2 (the goodness of fit can be found in Table S10). The best-fitted models for the removal and bioconcentration at 24 h were found for Tb (*R*^2^_adj_ = 0.998) and Y (*R*^2^_adj_ = 0.968), respectively, while at 96 h the best adjustments were observed for Gd (*R*^2^_adj_ = 0.941) and Tb (*R*^2^_adj_ = 0.968), for removal and bioconcentration, respectively.

**Table 2:** Reduced models of the removal (%) and bioconcentration (μg/g) responses as a function of the significant variables (*p-*value < 0.05) for *Ulva* sp.

	Removal (%)		Bioconcentration (μg/g)	
	24 h	96 h	24 h	96 h
Y	y = 61.6 − 2.07B + 4.91C + 8.24 × 10^−4^A^2^ + 2.66 × 10^−2^B^2^ + 8.14 × 10^−1^C^2^+ 1.28 × 10^−2^AC − 1.78 × 10^−1^BC	-	y = 116 + 1.55A – 2.58B – 57.3C – 3.31 × 10^−2^ AB – 9.17 × 10^−2^ + 8.37× 10^−1^ BC + 1.85 × 10^−3^ A^2^ + 5.49C^2^	-
La	y = 51.7 − 2.24 × 10^−1^A − 1.05B + 13.1C + 4.43 × 10^−4^A2 − 8.39 × 10^−1^C2 + 3.28 × 10^−3^AB	y = 65.1 − 5.06 × 10^−1^B + 5.67C − 1.20 × 10^−3^A^2^	y = 103 + 1.74A – 2.45B – 36.4C – 2.14 × 10^−1^AC + 6.06C^2^	y = 192 + 3.80A – 2.97B – 99.1C – 5.92 × 10^−1^AC + 16.0C^2^
Ce	y = 29.7 − 1.96 × 10^−1^A + 1.02B + 16.1C + 4.72 × 10^−4^A^2^ − 2.89 × 10^−2^B^2^ − 1.24C^2^	y = 75.1 − 1.88 × 10^−1^A + 11.3C + 4.87 × 10^−4^A^2^ − 1.24C^2^	y = 132 + 2.13A – 2.53B – 52.6C – 2.87 × 10^−1^AC + 8.68C^2^	y = 199 + 3.85A – 141C – 5.96 × 10^−1^AC +21.4C^2^
Eu	y = 60.9 − 2.16 × 10^−1^A − 1.14B + 8.89C + 3.57 × 10^−4^A2 + 4.51 × 10^−3^AB	y = 66.3 − 1.34 × 10^−1^A − 5.67 × 10^−1^B + 19.6C + 4.56 × 10^−4^A^2^ − 2.32C^2^	y = 132 + 1.98A – 2.61B – 53.5C – 2.47 × 10^−1^AC +8.58C^2^	y = 210 + 3.50A – 3.47B – 90C – 5.28× 10^−1^AC + 13.9C^2^
Gd	y = 59.3 − 1.26B + 9.10C + 4.99 × 10^−4^A^2^ + 4.48 × 10^−3^AB	y = 68.0 − 1.22B + 21.4C − 2.22C^2^ + 4.25 × 10^−3^AB − 1.73 × 10^−2^AC	y = -14.5 + 2.04A +3.07C – 2.69 × 10^−1^AC	y = 182 +3.51A – 3.29B – 86.4C – 5.42 × 10^−1^AC + 14.2C^2^
Tb	y = 54.5 − 2.37 × 10^−1^A − 7.64 × 10^−1^B + 8.76C + 6.03 × 10^−4^A^2^ − 1.25 × 10^−2^B^2^+ 4.22 × 10^−3^AB	y = 61.7 − 7.18 × 10^−1^B + 22.7C + 7.34 × 10^−4^A^2^ − 2.71C^2^	y = 101 + 1.66A – 2.7B – 38.1C – 2.11 × 10^−1^AC + 6.69C^2^	y = 289 – 3.11A – 8.91B - 109C – 4.79× 10^−1^AC +1.82BC + 10.7C^2^

Optimization studies focusing on the removal of La and Ce by seaweed have been reported in the literature (Keshtkar et al. 2019), however, with seaweed biomass after drying and grinding (not taking advantage of the bioaccumulation process), after functionalization treatment (increasing the cost and complexity of the process), and using central composite design (CCD) instead of BBD. Recent studies used RSM with a BBD to optimize Nd and Dy removal by two living seaweed (*Ulva* sp. and *Gracilaria* sp.), although both studies were performed with mono-element solutions (Fabre et al. 2021; Ferreira et al. 2021). Optimizing REEs removal from complex solutions by living seaweed is a new approach of the present study.

### Three-dimensional (3D) surface responses

**Fig. 3:**
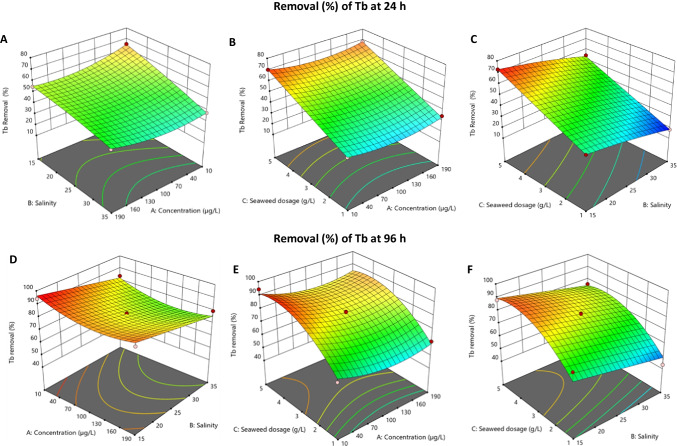
3D response surface for the removal (%) of Tb by *Ulva* sp. at 24 h and 96 h

**Fig. 4:**
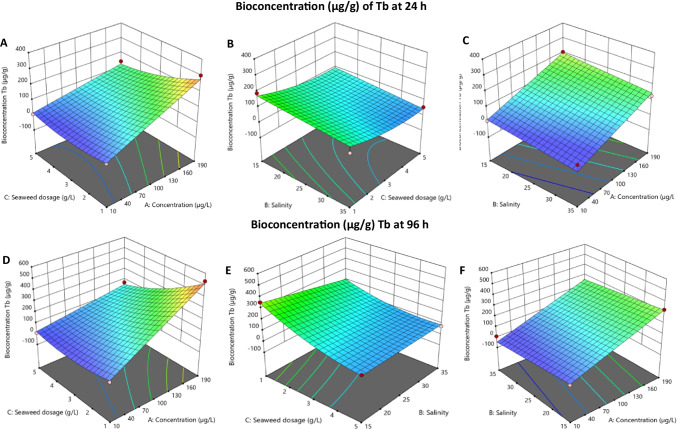
3D response surface for the bioconcentration (q_t_ (μg/g)) of Tb by *Ulva* sp. at 24 h and 96 h

Figures 3 and 4 show the three-dimensional (3D) response surfaces for Tb removal and bioconcentration by *Ulva* sp. at 24 and 96 h (the 3D response surfaces of the remaining REEs can be found in Supplementary material—Figure S1 to Figure S12). The interaction between salinity (B) and initial concentration (A) (Fig. 3A), at constant seaweed dosage (C = 3.0 g/L), shows that a higher removal is obtained when lower initial concentrations and salinities are applied for both elements, reaching removals close to 65 % for Tb. A positive effect on removal (up to 60 % of removal) is observed when the seaweed dosage increases and the initial concentration of Tb decreases, at constant salinity (B = 25) (Fig. 3B). Figure 3C shows that low salinity combined with an increase in seaweed dosage, while the initial concentration is kept constant (A = 100 μg/L), leads to higher removals. Extending the time to 96 h (Fig. 3D–F) increases the removal, but the way the variables interact is almost the same as discussed for 24 h.

In terms of bioconcentration (μg/g), the 3D surface plots for exposure times of 24 and 96 h are shown in Fig. 4. Seaweed dosage only affected bioconcentration at high concentrations of Tb (Fig. 4A), which may be because the number of active sites available on the seaweed surface is much higher than the number of REEs ions in solution (for low initial concentrations), resulting in low levels of REEs per g of seaweed, even if all the element in solution was bioaccumulated by *Ulva* sp. The results also showed that higher bioconcentration is achieved when a lower dosage of seaweed is applied at a higher concentration. Lower seaweed dosage in low salinity (Fig. 4B) shows higher bioconcentration of Tb, which may be attributed to less competition among ions in solution since there are fewer salt cations in solution at low salinity. At constant seaweed dosage (C = 3.0 g/L; Fig. 4C) bioconcentration increase is only dependent on the initial concentration, with a larger uptake occurring at higher concentrations at any salinity. Analyzing the uptake for 96 h (Fig. 4D – F), it is also possible to observe an enhancement in bioconcentration, as was observed for the removal, but the variables still interact in the same way as previously discussed for 24 h. As with removal, the results for the bioconcentration of the remaining REEs at 24 h and 96 h were consistent with those obtained for Tb.

### Optimal conditions for the removal of rare earth elements from the complex mixture

Optimizing the removal of some potentially toxic elements such as Pb, Cu, and Hg with *Ulva* sp. and *Gracilaria* sp. have been reported in the literature (Çetintaş et al. 2020; Isam et al. 2019), but all were performed after drying and grinding treatment of the seaweed biomass. For REEs, only studies for mono-solutions of Nd (Fabre et al. 2021) and Dy (Ferreira et al. 2021) using living seaweed are known. In the present study, RSM was applied to all REEs data to obtain the optimal conditions that maximize the removal of all REEs.

The optimization (Table 3) leads to removals from 67 % of Y to 88 % of Ce by *Ulva* sp. after 24 h under the operating conditions of 10 μg/L of REEs mixture, salinity of 15, and seaweed dosage of 5.0 g/L. After 96 h of exposure, the optimized response has values ≥ 90 % for all REEs under the operating conditions of 10 μg/L of REE mixture, salinity of 15, and seaweed dosage of 4.2 g/L. The highest removal is observed with the highest seaweed dosage, indicating that the available sorption sites are still unsaturated. Both conditions (high seaweed dosage and low salinity) appear to be consistent with previously information, as it is well described that increasing biomass dosage provides greater availability of binding sites, and consequently increases removal efficiency, while lower ionic strength reduces competition for binding sites (Cao et al. 2021).

**Table 3:** Optimal values for REEs removal (%) and bioconcentration (μg/g) with *Ulva* sp. and corresponding calculated bioconcentration (μg/g) or removal (%)

	REE	Time (h)	Initial concentration (μg/L)	Salinity	Seaweed dosage (g/L)	Removal (%)	Calculated bioconcentration (μg/g)
Optimized variables for REE removal (%)	Y	24	10	15	5.0	67	7.4
	La					79	8.7
	Ce					88	9.8
	Eu					87	9.6
	Gd					83	9.2
	Tb					84	9.4
	Y	96	10	15	4.2	-	-
	La					90	10
	Ce					99	11
	Eu					98	11
	Gd					100	11
	Tb					100	11
	REE	Time (h)	Initial concentration (μg/L)	Salinity	Seaweed dosage (g/L)	Bioconcentration (μg/g)	Calculated removal (%)
Optimized variables for REE bioconcentration (μg/g)	Y	24	190	15	1.0	253	24
	La					326	31
	Ce					401	38
	Eu					377	36
	Gd					325	31
	Tb					304	29
	Y	96	190	16	1.0	-	-
	La					668	63
	Ce					693	66
	Eu					667	63
	Gd					618	59
	Tb					576	55

The natural REE concentrations in seawater are usually very low (10^−6 ^– 10^−9^ g/L), increasing several order of magnitude in sites impacted by anthropogenic sources such as the discharge of industrial wastewater and e-waste leachates (Arienzo et al. 2022). For example, values of 130–152 μg REEs/L were reported in surface waters in areas affected by mining activities (Liang et al. 2014), which are in line with the range of concentrations of the present study, highlighting the potential of the approach to remediate contaminated environments. The use of living seaweed over non-living biomass can be valuable as the constant daily growth of seaweed creates new available binding sites, allowing for the removal of REEs from the complex solution even days after the onset of exposure (Costa et al. 2011; Henriques et al. 2015).

Bioconcentration (μg/g) optimization resulted in an initial element concentration of 190 μg/L, a salinity of 15, and a seaweed dosage 1.0 g/L, with values ranging from 253 μg/g for Y to 401 μg/g for Ce (∑REEs of 1986 μg/g), after 24 h exposure. For 96 h of exposure, a maximum ∑REEs of 3222 μg/g (excluding Y) was obtained, using the optimized conditions (initial concentration of 190 μg/L, salinity 16, and seaweed dosage of 1.0 g/L). The total concentration of REE in *Ulva* sp. is circa of 3000-fold higher than that initially in water and also exceeds that found in common apatite ores (∑REEs 1098 – 1688 μg/g) (Xiqiang et al. 2020), supporting the use of REEs-enriched seaweed biomass as an alternative to mineral ores. It is possible to further increase these concentrations by processing the biomass, namely its pyrolysis which reduces its weight by about 87 %.

The recovery of REEs from the biomass is also simpler than their extraction from ores which are much more difficult to solubilize. Diluted acids such as nitric and chloric acids or chelating agents have been used to regenerate various synthetic materials (Afonso et al. 2019; Lee et al. 2018; Ramasamy et al. 2017) and are also efficient in recovering REEs from seaweed. In some cases, the osmotic shock caused by placing the seaweed in ultrapure or distilled water, leading to disruption of the cell membrane, is enough for the elements to migrate into the solution (Henriques et al. 2019).

It should be noted that in addition to REEs, *Ulva* sp. also incorporated other elements, the separation of which is crucial for the valorization of technology. In a previous work, for the same time of exposure, we verified that REEs removed from water by *Ulva* sp. were mostly bound on the seaweed surface and were easily desorbed with a 15 min wash with EDTA (Viana et al. 2023). Other elements, such as the PTE Hg remained in the biomass after EDTA washing.

## Conclusion

The present study has demonstrated the potential of widely available seaweed as the basis of a simple, efficient, and low-cost technology to remove a myriad of elements from diluted industrial effluents and contaminated waters, especially those with high salinity that hamper the efficiency of other methods. The use of real saline water and a contamination scenario that mimics real effluent (e.g., from lamp production and dismantling) are also innovative aspects of this study.

Among the parameters studied, the initial seaweed dosage was the most impactful factor for REEs removal, with higher dosages resulting in greater removals (up to 88 % in 24 h). The time extension from 24 h to 96 or 144 h proved significant. Optimized conditions for REEs removal, obtained from the model optimization by RSM, were determined as a concentration of 10 μg/L at salinity 15 and seaweed dosage of 5.0 g/L.

The high REE concentration in the enriched biomass (∑REEs of 3222 μg/g) obtained under optimal conditions, which is approximately 3000 times higher than originally in the water and higher than in ordinary ores, encourages its use as an alternative source of these critical raw materials.

## Supplementary information

ESM 1 (DOCX 29255 kb)

## Data Availability

The datasets used or analyzed during the current study are available from the corresponding author on reasonable request.
