# Supplementary material for: Eco-friendly methodology for removing and recovering rare earth elements from saline industrial wastewater
Source: Environ Sci Pollut Res Int. 2023 Aug 14;30(42):96617–28. doi: 10.1007/s11356-023-29088-2 (PMC10482783; doi:10.1007/s11356-023-29088-2)
Supplement: Supplementary file 1 — (DOCX 29255 kb) [file 11356_2023_29088_MOESM1_ESM.docx]

**Supplementary material**

**Eco‑friendly methodology for removing and recovering rare earth elements from saline industrial wastewater**Thainara Viana^1^, Nicole Ferreira^1^, Daniela S. Tavares^1^, Azadeh Abdolvasei^1^, Eduarda Pereira^1,2^, Bruno Henriques^1*^

^1^LAQV-REQUIMTE - Associated Laboratory for Green Chemistry & Department of Chemistry, University of Aveiro, 3810-193 Aveiro, Portugal

^2^Central Laboratory of Analysis, University of Aveiro, 3810-193 Aveiro, Portugal

*Corresponding author: brunogalinho@ua.pt

**Methodology - Mercury quantification**

Quantification of Hg in solution was done by cold vapour atomic fluorescence spectrometry (CV-AFS) on a PSA cold vapour generator with a Merlin PSA detector. Tin chloride (SnCl_2_; 2 % w/v in 10 % v/v HCl) was used as the reducing agent. Standards with concentrations of 0.1, 0.2, 0.3, and 0.5 µg/L were used for the calibration curve. Quality control in the analysis was ensured by the assessment of each sample at least in triplicate, accepting results in which the coefficient of variation among reads was ≤ 10 %. The limit of quantification of the method was 0.02 μg/L. Quantification of Hg on the macroalgae tissue was performed by thermal decomposition atomic absorption spectrometry with gold amalgamation (TD-AAS - LECO model AMA-254). The analysis was made directly on the sample (1 - 20 mg dry weight (DW)), without digestion or specific pre-treatment. Analytical quality control was performed by using certified reference materials: BCR-060 - Aquatic Plant (*Lagarosiphon major*) [Hg] = 0.34 ± 0.04 mg/kg and Dolt-3 - Dogfish Liver [Hg] = 3.37 ± 0.04 mg/kg, which were analysed every day before the beginning of the analysis and repeated at the end of the day. All percentages of recovery for total Hg were within the range of 105 – 111 %.

**Table S1:** Experimental design matrix describing each experimental condition (A – element concentration in solution; B – salinity; C – seaweed dosage).

| Variables | | |  | Levels | | |
| --- | --- | --- | --- | --- | --- | --- |
|  |  |  |  | -1 | 0 | 1 |
| A - Initial concentration (µg/L) | | |  | 10 | 100 | 190 |
| B - Salinity |  |  |  | 15 | 25 | 35 |
| C - Seaweed dosage (g/L) | | |  | 1 | 3 | 5 |
| Coded and uncoded level of variables | | | | | | |
| Experiments |  | A |  | B |  | C |
| 1 |  | 10 (-1) |  | 25 (0) |  | 1.0 (-1) |
| 2 |  | 190 (1) |  | 25 (0) |  | 1.0 (-1) |
| 3 |  | 10 (-1) |  | 25 (0) |  | 5.0 (1) |
| 4 |  | 190 (1) |  | 25 (0) |  | 5.0 (1) |
| 5 |  | 100 (0) |  | 15 (-1) |  | 1.0 (-1) |
| 6 |  | 100 (0) |  | 35 (1) |  | 1.0 (-1) |
| 7 |  | 100 (0) |  | 15 (-1) |  | 5.0 (1) |
| 8 |  | 100 (0) |  | 35 (1) |  | 5.0 (1) |
| 9 |  | 10 (-1) |  | 15 (-1) |  | 3.0 (0) |
| 10 |  | 10 (-1) |  | 35 (1) |  | 3.0 (0) |
| 11 |  | 190 (1) |  | 15 (-1) |  | 3.0 (0) |
| 12 |  | 190 (1) |  | 35 (1) |  | 3.0 (0) |
| 13 |  | 100 (0) |  | 25 (0) |  | 3.0 (0) |
| 14 |  | 100 (0) |  | 25 (0) |  | 3.0 (0) |
| 15 |  | 100 (0) |  | 25 (0) |  | 3.0 (0) |

**Table S2:** Friedman´s test with Dunn's multiple comparison test applied to the element’s removal (%) and element´s bioconcentration (µg/g) by Ulva sp. from the mixture mimicking the elemental composition of industrial effluent, at different exposure times.

| **Exposure time** | **Friedman’s test with Dunn's multiple comparison test (*p*-value < 0.05)** | | | | | | | | | |
| --- | --- | --- | --- | --- | --- | --- | --- | --- | --- | --- |
|  | Removal (%) | | | | | bioconcentration (µg/g) | | | | |
|  | **La** | **Ce** | **Eu** | **Gd** | **Tb** | **La** | **Ce** | **Eu** | **Gd** | **Tb** |
| **24 h vs 48 h** | 0.2034 | 0.2034 | 0.3960 | 0.3960 | 0.3960 | 0.2034 | 0.1419 | 0.3960 | 0.3960 | 0.5381 |
| **24 h vs 96 h** | <0.0001 | <0.0001 | 0.0002 | 0.0002 | 0.0002 | <0.0001 | <0.0001 | 0.0002 | <0.0001 | 0.0002 |
|  |  |  |  |  |  |  |  |  |  |  |
| **24 h vs 144 h** | <0.0001 | <0.0001 | <0.0001 | <0.0001 | <0.0001 | <0.0001 | <0.0001 | <0.0001 | <0.0001 | <0.0001 |
|  |  |  |  |  |  |  |  |  |  |  |
| **48 h vs 96 h** | 0.1703 | 0.1419 | 0.1177 | 0.1177 | 0.1177 | 0.1703 | 0.1419 | 0.1177 | 0.0800 | 0.0800 |
| **48 h vs 144 h** | 0.0002 | 0.0002 | <0.0001 | <0.0001 | <0.0001 | 0.0002 | 0.0014 | <0.0001 | 0.0002 | <0.0001 |
|  |  |  |  |  |  |  |  |  |  |  |
| **96 h vs 144 h** | 0.2863 | 0.3960 | 0.2863 | 0.2863 | 0.2863 | 0.2863 | 0.9438 | 0.2863 | 0.5381 | 0.3960 |

**Table S3:** Bioconcentration factor for REEs after 144 h of exposure to BBD conditions.

| Trials | BCF | | | | | |
| --- | --- | --- | --- | --- | --- | --- |
|  | Y | La | Ce | Eu | Gd | Tb |
| 1 | 2600 | 4300 | 4800 | 4500 | 4300 | 3100 |
| 2 | 3711 | 4453 | 4453 | 4032 | 4084 | 3553 |
| 3 | - | 1300 | 1300 | 1300 | 1200 | 1000 |
| 4 | 1137 | 1221 | 1232 | 1116 | 1126 | 963 |
| 5 | 4320 | 4780 | 5350 | 4940 | 4480 | 4010 |
| 6 | 2030 | 3950 | 4560 | 3750 | 3440 | 2730 |
| 7 | 1050 | 1140 | 1250 | 1150 | 1160 | 970 |
| 8 | 1010 | 1090 | 1160 | 1090 | 1040 | 900 |
| 9 | - | 2100 | 2100 | 2100 | 1900 | 1700 |
| 10 | - | 1900 | 2000 | 1800 | 1900 | 1300 |
| 11 | 2005 | 2163 | 2263 | 2174 | 1984 | 1816 |
| 12 | 1474 | 1458 | 1458 | 1458 | 1400 | 1253 |
| 13 | 1490 | 1620 | 1710 | 1580 | 1490 | 1310 |
| 14 | 1510 | 1640 | 1710 | 1610 | 1520 | 1330 |
| 15 | 1610 | 1770 | 1880 | 1740 | 1650 | 1400 |

**Table S4**: ANOVA values for quadratic model parameters of Y in the removal (%) response.

| **Y Removal (%)** | **24 h** | | | | **48 h** | | | |
| --- | --- | --- | --- | --- | --- | --- | --- | --- |
|  | **df** | **Mean square** | **F-value** | **p-value** | **df** | **Mean square** | **F-value** | **p-value** |
| **Model** | 9 | 330.78 | 176.91 | < 0.0001 | 9 | 362.01 | 15.34 | 0.0039 |
| **A** | 1 | 0.0141 | 0.0075 | 0.9342 | 1 | 3.80 | 0.1608 | 0.7050 |
| **B** | 1 | 1298.83 | 694.67 | < 0.0001 | 1 | 1956.29 | 82.87 | 0.0003 |
| **C** | 1 | 1399.42 | 748.47 | < 0.0001 | 1 | 794.87 | 33.67 | 0.0021 |
| **AB** | 1 | 0.0664 | 0.0355 | 0.8579 | 1 | 2.18 | 0.0923 | 0.7735 |
| **AC** | 1 | 21.17 | 11.32 | 0.0200 | 1 | 8.55 | 0.3622 | 0.5735 |
| **BC** | 1 | 50.85 | 27.20 | 0.0034 | 1 | 41.17 | 1.74 | 0.2439 |
| **A^2^** | 1 | 164.46 | 87.96 | 0.0002 | 1 | 304.01 | 12.88 | 0.0157 |
| **B^2^** | 1 | 26.16 | 13.99 | 0.0134 | 1 | 8.92 | 0.3780 | 0.5656 |
| **C^2^** | 1 | 39.14 | 20.94 | 0.0060 | 1 | 104.80 | 4.44 | 0.0890 |
| **Residual** | 5 | 1.87 |  |  | 5 | 23.61 |  |  |
| **Lack of fit** | 3 | 3.05 | 29.93 | 0.0325 | 3 | 24.35 | 1.08 | 0.5132 |
| **Pure error** | 2 | 0.1018 |  |  | 2 | 22.50 |  |  |
|  |  | **R^2^** | 0.9969 |  |  | **R^2^** | 0.9650 |  |
|  |  | **R^2^ adj** | 0.9912 |  |  | **R^2^ adj** | 0.9021 |  |
|  |  | **R^2^ pred** | 0.9509 |  |  | **R^2^ pred** | 0.6239 |  |
|  |  | **Adeq. Precision** | 465183 |  |  | **Adeq. Precision** | 132303 |  |

**Table S5**: ANOVA values for quadratic model parameters of La in the removal (%) response.

| **La Removal (%)** | **24 h** | | | | **48 h** | | | | **96 h** | | | | **144 h** | | | |
| --- | --- | --- | --- | --- | --- | --- | --- | --- | --- | --- | --- | --- | --- | --- | --- | --- |
|  | **df** | **Mean square** | **F-value** | **p-value** | **df** | **Mean square** | **F-value** | **p-value** | **df** | **Mean square** | **F-value** | **p-value** | **df** | **Mean square** | **F-value** | **p-value** |
| **Model** | 9 | 312.77 | 115.36 | < 0.0001 | 9 | 287.28 | 36.20 | 0.0005 | 9 | 186.03 | 42.41 | 0.0003 | 9 | 100.77 | 9.57 | 0.0114 |
| **A** | 1 | 185.94 | 68.58 | 0.0004 | 1 | 21.26 | 2.68 | 0.1626 | 1 | 25.36 | 5.78 | 0.0613 | 1 | 28.87 | 2.74 | 0.1586 |
| **B** | 1 | 418.50 | 154.36 | < 0.0001 | 1 | 577.03 | 72.70 | 0.0004 | 1 | 204.81 | 46.69 | 0.0010 | 1 | 87.81 | 8.34 | 0.0343 |
| **C** | 1 | 2066.96 | 762.37 | < 0.0001 | 1 | 1588.56 | 200.15 | < 0.0001 | 1 | 1029.50 | 234.70 | < 0.0001 | 1 | 564.90 | 53.65 | 0.0007 |
| **AB** | 1 | 34.88 | 12.86 | 0.0158 | 1 | 68.25 | 8.60 | 0.0325 | 1 | 3.23 | 0.7367 | 0.4299 | 1 | 17.39 | 1.65 | 0.2550 |
| **AC** | 1 | 2.99 | 1.10 | 0.3416 | 1 | 14.64 | 1.84 | 0.2325 | 1 | 25.76 | 5.87 | 0.0599 | 1 | 70.41 | 6.69 | 0.0491 |
| **BC** | 1 | 2.33 | 0.8579 | 0.3968 | 1 | 18.96 | 2.39 | 0.1829 | 1 | 5.51 | 1.26 | 0.3135 | 1 | 5.85 | 0.5554 | 0.4896 |
| **A^2^** | 1 | 44.88 | 16.55 | 0.0096 | 1 | 67.84 | 8.55 | 0.0329 | 1 | 22.44 | 5.12 | 0.0732 | 1 | 14.08 | 1.34 | 0.2997 |
| **B^2^** | 1 | 6.71 | 2.48 | 0.1764 | 1 | 15.42 | 1.94 | 0.2222 | 1 | 5.88 | 1.34 | 0.2991 | 1 | 18.14 | 1.72 | 0.2463 |
| **C^2^** | 1 | 44.19 | 16.30 | 0.0099 | 1 | 197.05 | 24.83 | 0.0042 | 1 | 330.61 | 75.37 | 0.0003 | 1 | 89.49 | 8.50 | 0.0332 |
| **Residual** | 5 | 2.71 |  |  | 5 | 7.94 |  |  | 5 | 4.39 |  |  | 5 | 10.53 |  |  |
| **Lack of fit** | 3 | 2.83 | 1.11 | 0.5055 | 3 | 8.85 | 1.35 | 0.4529 | 3 | 4.90 | 1.36 | 0.4505 | 3 | 17.22 | 34.54 | 0.0283 |
| **Pure error** | 2 | 2.54 |  |  | 2 | 6.57 |  |  | 2 | 3.61 |  |  | 2 | 0.4985 |  |  |
|  |  | **R^2^** | 0.9952 |  |  | **R^2^** | 0.9849 |  |  | **R^2^** | 0.9871 |  |  | **R^2^** | 0.9451 |  |
|  |  | **R^2^ adj** | 0.9866 |  |  | **R^2^ adj** | 0.9577 |  |  | **R^2^ adj** | 0.9638 |  |  | **R^2^ adj** | 0.8464 |  |
|  |  | **R^2^ pred** | 0.9480 |  |  | **R^2^ pred** | 0.8270 |  |  | **R^2^ pred** | 0.8516 |  |  | **R^2^ pred** | 0.1365 |  |
|  |  | **Adeq. Precision** | 365501 |  |  | **Adeq. Precision** | 201077 |  |  | **Adeq. Precision** | 217794 |  |  | **Adeq. Precision** | 102513 |  |

**Table S6**: ANOVA values for quadratic model parameters of Ce in the removal (%) response.

| **Ce Removal (%)** | **24 h** | | | | **48 h** | | | | **96 h** | | | | **144 h** | | | |
| --- | --- | --- | --- | --- | --- | --- | --- | --- | --- | --- | --- | --- | --- | --- | --- | --- |
|  | **df** | **Mean square** | **F-value** | **p-value** | **df** | **Mean square** | **F-value** | **p-value** | **df** | **Mean square** | **F-value** | **p-value** | **df** | **Mean square** | **F-value** | **p-value** |
| **Model** | 9 | 382.37 | 95.21 | < 0.0001 | 9 | 343.33 | 43.87 | 0.0003 | 9 | 137.67 | 17.49 | 0.0029 | 9 | 61.09 | 8.94 | 0.0133 |
| **A** | 1 | 670.28 | 166.90 | < 0.0001 | 1 | 776.94 | 99.27 | 0.0002 | 1 | 526.83 | 66.93 | 0.0004 | 1 | 226.37 | 33.13 | 0.0022 |
| **B** | 1 | 144.40 | 35.96 | 0.0019 | 1 | 72.99 | 9.33 | 0.0283 | 1 | 18.40 | 2.34 | 0.1868 | 1 | 32.00 | 4.68 | 0.0828 |
| **C** | 1 | 2399.40 | 597.44 | < 0.0001 | 1 | 1814.99 | 231.90 | < 0.0001 | 1 | 467.89 | 59.44 | 0.0006 | 1 | 206.45 | 30.21 | 0.0027 |
| **AB** | 1 | 14.22 | 3.54 | 0.1186 | 1 | 0.0002 | 0.0000 | 0.9966 | 1 | 10.31 | 1.31 | 0.3042 | 1 | 31.18 | 4.56 | 0.0857 |
| **AC** | 1 | 17.95 | 4.47 | 0.0882 | 1 | 1.47 | 0.1882 | 0.6825 | 1 | 37.44 | 4.76 | 0.0810 | 1 | 1.71 | 0.2507 | 0.6378 |
| **BC** | 1 | 8.95 | 2.23 | 0.1957 | 1 | 3.86 | 0.4930 | 0.5139 | 1 | 0.002915 | 0.00037 | 0.9995 | 1 | 0.1163 | 0.0170 | 0.9013 |
| **A^2^** | 1 | 54.09 | 13.47 | 0.0144 | 1 | 105.22 | 13.44 | 0.0145 | 1 | 62.55 | 7.95 | 0.0372 | 1 | 31.41 | 4.60 | 0.0849 |
| **B^2^** | 1 | 30.90 | 7.69 | 0.0392 | 1 | 10.39 | 1.33 | 0.3014 | 1 | 17.80 | 2.26 | 0.1929 | 1 | 5.07 | 0.7419 | 0.4284 |
| **C^2^** | 1 | 90.80 | 22.61 | 0.0051 | 1 | 278.03 | 35.52 | 0.0019 | 1 | 84.71 | 10.76 | 0.0219 | 1 | 12.77 | 1.87 | 0.2299 |
| **Residual** | 5 | 4.02 |  |  | 5 | 7.83 |  |  | 5 | 7.87 |  |  | 5 | 6.83 |  |  |
| **Lack of fit** | 3 | 5.15 | 2.22 | 0.3253 | 3 | 7.33 | 0.8565 | 0.5783 | 3 | 9.68 | 1.88 | 0.3663 | 3 | 10.84 | 13.28 | 0.0708 |
| **Pure error** | 2 | 2.32 |  |  | 2 | 8.56 |  |  | 2 | 5.16 |  |  | 2 | 0.8164 |  |  |
|  |  | **R^2^** | 0.9942 |  |  | **R^2^** | 0.9875 |  |  | **R^2^** | 0.9692 |  |  | **R^2^** | 0.9415 |  |
|  |  | **R^2^ adj** | 0.9838 |  |  | **R^2^ adj** | 0.9650 |  |  | **R^2^ adj** | 0.9138 |  |  | **R^2^ adj** | 0.8362 |  |
|  |  | **R^2^ pred** | 0.9256 |  |  | **R^2^ pred** | 0.8752 |  |  | **R^2^ pred** | 0.6184 |  |  | **R^2^ pred** | 0.1024 |  |
|  |  | **Adeq. Precision** | 356743 |  |  | **Adeq. Precision** | 220603 |  |  | **Adeq. Precision** | 148474 |  |  | **Adeq. Precision** | 97448 |  |

**Table S7**: ANOVA values for quadratic model parameters of Eu in the removal (%) response.

| **Eu Removal (%)** | **24 h** | | | | **48 h** | | | | **96 h** | | | | **144 h** | | | | |
| --- | --- | --- | --- | --- | --- | --- | --- | --- | --- | --- | --- | --- | --- | --- | --- | --- | --- |
|  | **df** | **Mean square** | **F-value** | **p-value** | **df** | **Mean square** | **F-value** | **p-value** | **df** | **Mean square** | **F-value** | **p-value** | **df** | **Mean square** | **F-value** | **p-value** |  |
| **Model** | 9 | 343.65 | 150.48 | < 0.0001 | 9 | 311.34 | 88.39 | < 0.0001 | 9 | 208.40 | 38.69 | 0.0004 | 9 | 107.28 | 24.29 | 0.0013 |  |
| **A** | 1 | 67.26 | 29.45 | 0.0029 | 1 | 6.06 | 1.72 | 0.2467 | 1 | 121.25 | 22.51 | 0.0051 | 1 | 86.91 | 19.68 | 0.0068 |  |
| **B** | 1 | 384.57 | 168.40 | < 0.0001 | 1 | 927.70 | 263.39 | < 0.0001 | 1 | 257.56 | 47.81 | 0.0010 | 1 | 105.20 | 23.82 | 0.0045 |  |
| **C** | 1 | 2531.18 | 1108.35 | < 0.0001 | 1 | 1564.92 | 444.31 | < 0.0001 | 1 | 1056.92 | 196.20 | < 0.0001 | 1 | 540.27 | 122.34 | 0.0001 |  |
| **AB** | 1 | 65.94 | 28.88 | 0.0030 | 1 | 61.35 | 17.42 | 0.0087 | 1 | 1.06 | 0.1972 | 0.6756 | 1 | 21.71 | 4.92 | 0.0774 |  |
| **AC** | 1 | 2.41 | 1.06 | 0.3513 | 1 | 21.66 | 6.15 | 0.0559 | 1 | 23.70 | 4.40 | 0.0900 | 1 | 38.71 | 8.77 | 0.0315 |  |
| **BC** | 1 | 0.9534 | 0.4175 | 0.5467 | 1 | 7.59 | 2.15 | 0.2021 | 1 | 14.13 | 2.62 | 0.1662 | 1 | 26.99 | 6.11 | 0.0564 |  |
| **A^2^** | 1 | 27.43 | 12.01 | 0.0179 | 1 | 76.43 | 21.70 | 0.0055 | 1 | 54.02 | 10.03 | 0.0249 | 1 | 32.74 | 7.41 | 0.0416 |  |
| **B^2^** | 1 | 3.99 | 1.75 | 0.2437 | 1 | 6.01 | 1.71 | 0.2483 | 1 | 10.84 | 2.01 | 0.2152 | 1 | 21.64 | 4.90 | 0.0777 |  |
| **C^2^** | 1 | 6.03 | 2.64 | 0.1651 | 1 | 115.30 | 32.73 | 0.0023 | 1 | 308.46 | 57.26 | 0.0006 | 1 | 80.24 | 18.17 | 0.0080 |  |
| **Residual** | 5 | 2.28 |  |  | 5 | 3.52 |  |  | 5 | 5.39 |  |  | 5 | 4.42 |  |  |  |
| **Lack of fit** | 3 | 2.44 | 1.20 | 0.4856 | 3 | 3.09 | 0.7390 | 0.6188 | 3 | 7.97 | 5.28 | 0.1633 | 3 | 6.79 | 7.90 | 0.1144 |  |
| **Pure error** | 2 | 2.04 |  |  | 2 | 4.18 |  |  | 2 | 1.51 |  |  | 2 | 0.8592 |  |  |  |
|  |  | **R^2^** | 0.9963 |  |  | **R^2^** | 0.9938 |  |  | **R^2^** | 0.9858 |  |  | **R^2^** | 0.9776 |  |  |
|  |  | **R^2^ adj** | 0.9897 |  |  | **R^2^ adj** | 0.9825 |  |  | **R^2^ adj** | 0.9604 |  |  | **R^2^ adj** | 0.9374 |  |  |
|  |  | **R^2^ pred** | 0.9593 |  |  | **R^2^ pred** | 0.9408 |  |  | **R^2^ pred** | 0.7953 |  |  | **R^2^ pred** | 0.6662 |  |  |
|  |  | **Adeq. Precision** | 408764 |  |  | **Adeq. Precision** | 323095 |  |  | **Adeq. Precision** | 222129 |  |  | **Adeq. Precision** | 172651 |  |  |

**Table S8**: ANOVA values for quadratic model parameters of Gd in the removal (%) response.

| **Gd Removal (%)** | **24 h** | | | | **48 h** | | | | **96 h** | | | | **144 h** | | | |
| --- | --- | --- | --- | --- | --- | --- | --- | --- | --- | --- | --- | --- | --- | --- | --- | --- |
|  | **df** | **Mean square** | **F-value** | **p-value** | **df** | **Mean square** | **F-value** | **p-value** | **df** | **Mean square** | **F-value** | **p-value** | **df** | **Mean square** | **F-value** | **p-value** |
| **Model** | 9 | 371.24 | 85.35 | < 0.0001 | 9 | 337.76 | 237.42 | < 0.0001 | 9 | 249.95 | 51.14 | 0.0002 | 9 | 115.67 | 20.53 | 0.0020 |
| **A** | 1 | 16.86 | 3.88 | 0.1060 | 1 | 3.44 | 2.42 | 0.1807 | 1 | 13.54 | 2.77 | 0.1569 | 1 | 67.88 | 12.05 | 0.0178 |
| **B** | 1 | 531.26 | 122.14 | 0.0001 | 1 | 1057.46 | 743.31 | < 0.0001 | 1 | 508.40 | 104.02 | 0.0002 | 1 | 110.71 | 19.65 | 0.0068 |
| **C** | 1 | 2650.10 | 609.28 | < 0.0001 | 1 | 1653.70 | 1162.42 | < 0.0001 | 1 | 1288.78 | 263.69 | < 0.0001 | 1 | 620.79 | 110.17 | 0.0001 |
| **AB** | 1 | 65.08 | 14.96 | 0.0118 | 1 | 60.30 | 42.39 | 0.0013 | 1 | 58.68 | 12.01 | 0.0179 | 1 | 20.82 | 3.69 | 0.1126 |
| **AC** | 1 | 0.2391 | 0.0550 | 0.8239 | 1 | 18.59 | 13.07 | 0.0153 | 1 | 38.90 | 7.96 | 0.0371 | 1 | 46.77 | 8.30 | 0.0346 |
| **BC** | 1 | 0.0983 | 0.0226 | 0.8864 | 1 | 10.60 | 7.45 | 0.0413 | 1 | 21.38 | 4.37 | 0.0907 | 1 | 29.13 | 5.17 | 0.0721 |
| **A^2^** | 1 | 55.78 | 12.82 | 0.0159 | 1 | 85.20 | 59.89 | 0.0006 | 1 | 25.54 | 5.22 | 0.0710 | 1 | 43.43 | 7.71 | 0.0391 |
| **B^2^** | 1 | 16.43 | 3.78 | 0.1096 | 1 | 11.12 | 7.82 | 0.0382 | 1 | 0.1519 | 0.0311 | 0.8670 | 1 | 16.41 | 2.91 | 0.1486 |
| **C^2^** | 1 | 0.0486 | 0.0112 | 0.9200 | 1 | 123.11 | 86.54 | 0.0002 | 1 | 279.25 | 57.14 | 0.0006 | 1 | 74.24 | 13.18 | 0.0151 |
| **Residual** | 5 | 4.35 |  |  | 5 | 1.42 |  |  | 5 | 4.89 |  |  | 5 | 5.63 |  |  |
| **Lack of fit** | 3 | 7.04 | 22.00 | 0.0438 | 3 | 0.5570 | 0.2047 | 0.8861 | 3 | 7.89 | 20.77 | 0.0463 | 3 | 8.80 | 9.88 | 0.0933 |
| **Pure error** | 2 | 0.3199 |  |  | 2 | 2.72 |  |  | 2 | 0.3799 |  |  | 2 | 0.8901 |  |  |
|  |  | **R^2^** | 0.9935 |  |  | **R^2^** | 0.9977 |  |  | **R^2^** | 0.9893 |  |  | **R^2^** | 0.9737 |  |
|  |  | **R^2^ adj** | 0.9819 |  |  | **R^2^ adj** | 0.9935 |  |  | **R^2^ adj** | 0.9699 |  |  | **R^2^ adj** | 0.9262 |  |
|  |  | **R^2^ pred** | 0.8991 |  |  | **R^2^ pred** | 0.9872 |  |  | **R^2^ pred** | 0.8326 |  |  | **R^2^ pred** | 0.6013 |  |
|  |  | **Adeq. Precision** | 309478 |  |  | **Adeq. Precision** | 531377 |  |  | **Adeq. Precision** | 262621 |  |  | **Adeq. Precision** | 163509 |  |

**Table S9**: ANOVA values for quadratic model parameters of Tb in the removal (%) response.

| **Tb Removal (%)** | **24 h** | | | | **48 h** | | | | **96 h** | | | | **144 h** | | | | |
| --- | --- | --- | --- | --- | --- | --- | --- | --- | --- | --- | --- | --- | --- | --- | --- | --- | --- |
|  | **df** | **Mean square** | **F-value** | **p-value** | **df** | **Mean square** | **F-value** | **p-value** | **df** | **Mean square** | **F-value** | **p-value** | **df** | **Mean square** | **F-value** | **p-value** |  |
| **Model** | 9 | 374.70 | 1055.08 | < 0.0001 | 9 | 360.06 | 48.94 | 0.0002 | 9 | 279.33 | 23.36 | 0.0014 | 9 | 142.36 | 21.37 | 0.0018 |  |
| **A** | 1 | 7.37 | 20.75 | 0.0061 | 1 | 0.0893 | 0.0121 | 0.9165 | 1 | 76.39 | 6.39 | 0.0527 | 1 | 65.19 | 9.79 | 0.0260 |  |
| **B** | 1 | 751.27 | 2115.42 | < 0.0001 | 1 | 1358.11 | 184.61 | < 0.0001 | 1 | 412.60 | 34.51 | 0.0020 | 1 | 152.20 | 22.85 | 0.0050 |  |
| **C** | 1 | 2455.90 | 6915.33 | < 0.0001 | 1 | 1447.31 | 196.74 | < 0.0001 | 1 | 1331.62 | 111.37 | 0.0001 | 1 | 712.79 | 107.00 | 0.0001 |  |
| **AB** | 1 | 57.67 | 162.38 | < 0.0001 | 1 | 44.52 | 6.05 | 0.0572 | 1 | 1.95 | 0.1631 | 0.7030 | 1 | 19.79 | 2.97 | 0.1454 |  |
| **AC** | 1 | 0.7454 | 2.10 | 0.2071 | 1 | 21.73 | 2.95 | 0.1463 | 1 | 28.53 | 2.39 | 0.1831 | 1 | 49.47 | 7.43 | 0.0415 |  |
| **BC** | 1 | 0.2998 | 0.8441 | 0.4004 | 1 | 10.42 | 1.42 | 0.2875 | 1 | 58.29 | 4.88 | 0.0783 | 1 | 73.46 | 11.03 | 0.0210 |  |
| **A^2^** | 1 | 89.43 | 251.80 | < 0.0001 | 1 | 165.94 | 22.56 | 0.0051 | 1 | 131.89 | 11.03 | 0.0210 | 1 | 65.30 | 9.80 | 0.0259 |  |
| **B^2^** | 1 | 5.47 | 15.40 | 0.0111 | 1 | 29.15 | 3.96 | 0.1032 | 1 | 0.5429 | 0.0454 | 0.8397 | 1 | 11.35 | 1.70 | 0.2486 |  |
| **C^2^** | 1 | 0.8619 | 2.43 | 0.1800 | 1 | 136.87 | 18.61 | 0.0076 | 1 | 430.54 | 36.01 | 0.0018 | 1 | 115.74 | 17.37 | 0.0088 |  |
| **Residual** | 5 | 0.3551 |  |  | 5 | 7.36 |  |  | 5 | 11.96 |  |  | 5 | 6.66 |  |  |  |
| **Lack of fit** | 3 | 0.5919 |  |  | 3 | 3.85 | 0.3049 | 0.8242 | 3 | 19.12 | 15.83 | 0.0600 | 3 | 10.95 | 46.71 | 0.0210 |  |
| **Pure error** | 2 | 0.0000 |  |  | 2 | 12.62 |  |  | 2 | 1.21 |  |  | 2 | 0.2344 |  |  |  |
|  |  | **R^2^** | 0.9995 |  |  | **R^2^** | 0.9888 |  |  | **R^2^** | 0.9768 |  |  | **R^2^** | 0.9747 |  |  |
|  |  | **R^2^ adj** | 0.9985 |  |  | **R^2^ adj** | 0.9686 |  |  | **R^2^ adj** | 0.9350 |  |  | **R^2^ adj** | 0.9291 |  |  |
|  |  | **R^2^ pred** | 0.9916 |  |  | **R^2^ pred** | 0.9263 |  |  | **R^2^ pred** | 0.6413 |  |  | **R^2^ pred** | 0.5995 |  |  |
|  |  | **Adeq. Precision** | 1118491 |  |  | **Adeq. Precision** | 243469 |  |  | **Adeq. Precision** | 182923 |  |  | **Adeq. Precision** | 172487 |  |  |

**Table S10:** Comparison of the goodness of fit obtained by the response surface methodology, for removal (%) and bioconcentration (µg/g).

| REEs | Removal (%) | | Bioconcentration (µg/g) | |  |
| --- | --- | --- | --- | --- | --- |
|  | R^2^_adj_ | | R^2^_adj_ | |  |
|  | 24 h | 96 h | 24 h | 96 h |  |
| **Y** | 0.994 | - | 0.968 | - |  |
|  |  |  |  |  |  |
| **La** | 0.984 | 0.919 | 0.941 | 0.963 |  |
|  |  |  |  |  |  |
| **Ce** | 0.969 | 0.865 | 0.935 | 0.940 |  |
|  |  |  |  |  |  |
| **Eu** | 0.988 | 0.937 | 0.943 | 0.957 |  |
|  |  |  |  |  |  |
| **Gd** | 0.977 | 0.941 | 0.835 | 0.958 |  |
|  |  |  |  |  |  |
| **Tb** | 0.998 | 0.877 | 0.911 | 0.968 |  |
|  |  |  |  |  |  |

**F**


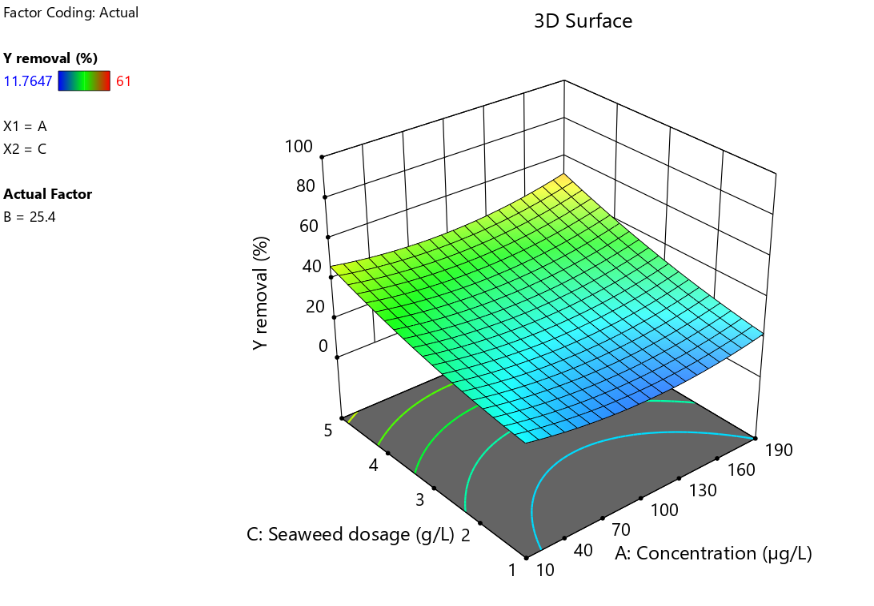


**Removal (%) of Y at 24 h**

**C**

**B**

**A**


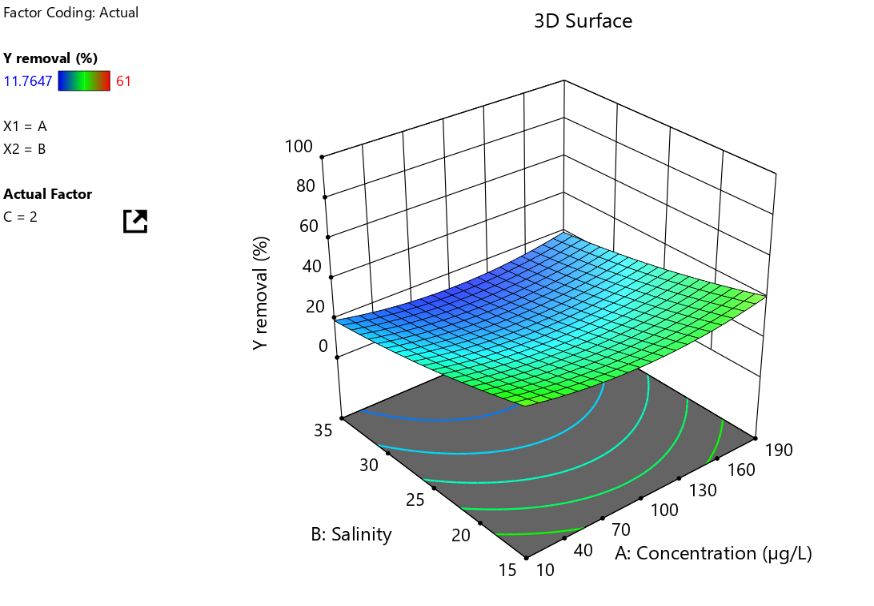

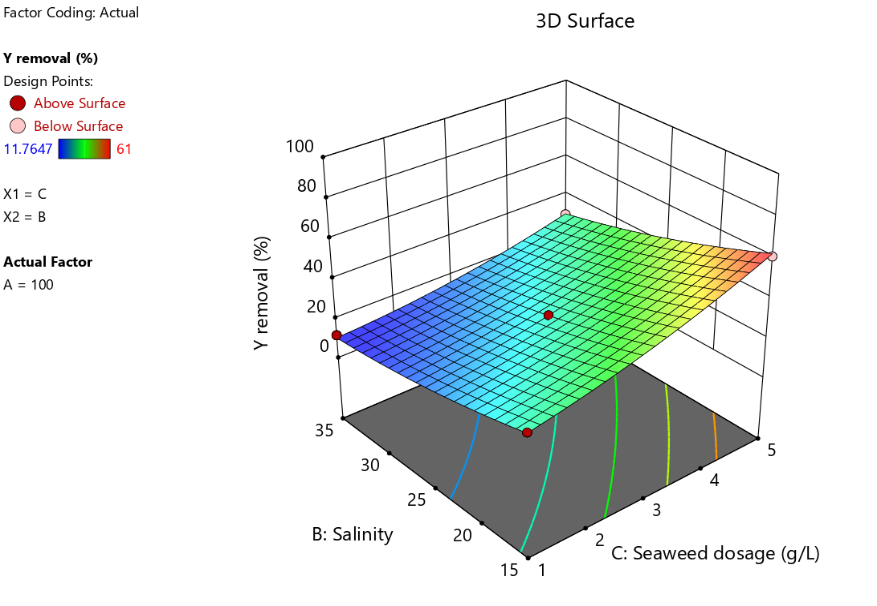

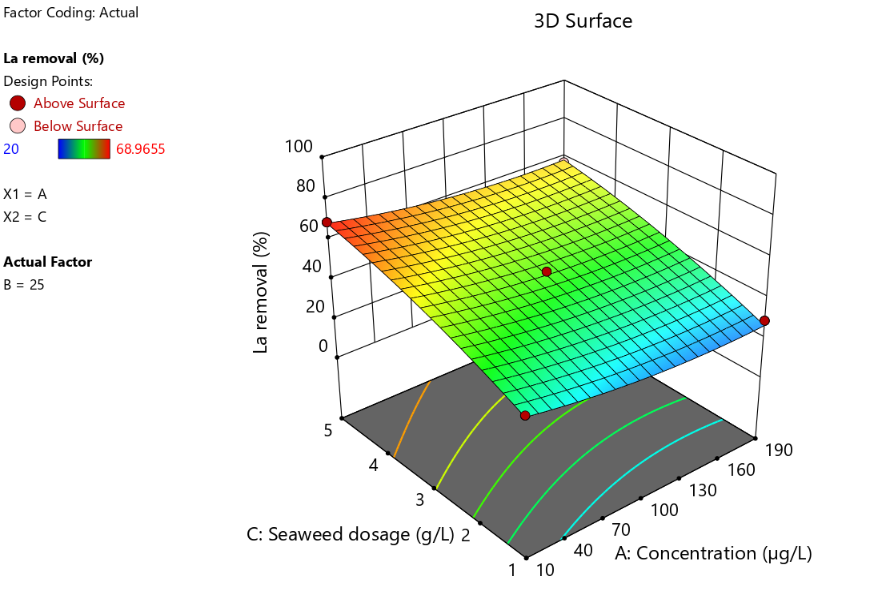


**Removal (%) of La at 24 h**

**E**

**D**

**F**


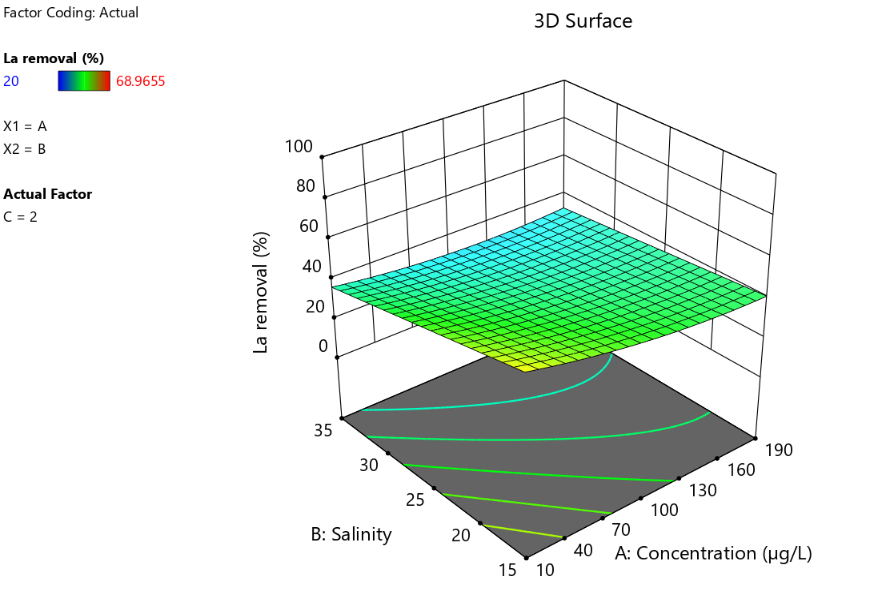

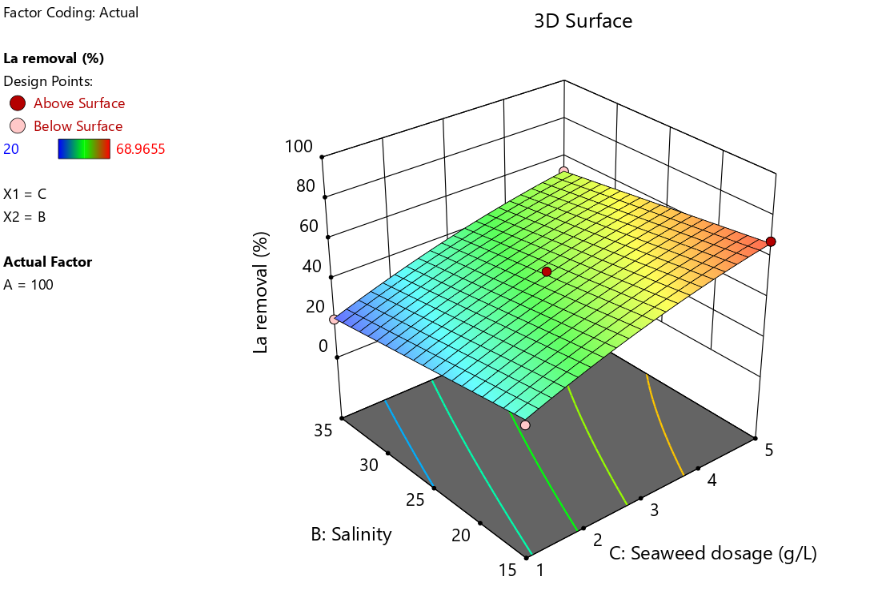


**Figure S1**: 3D response surface for the removal (%) of Y and La by *Ulva* sp. at 24 h.


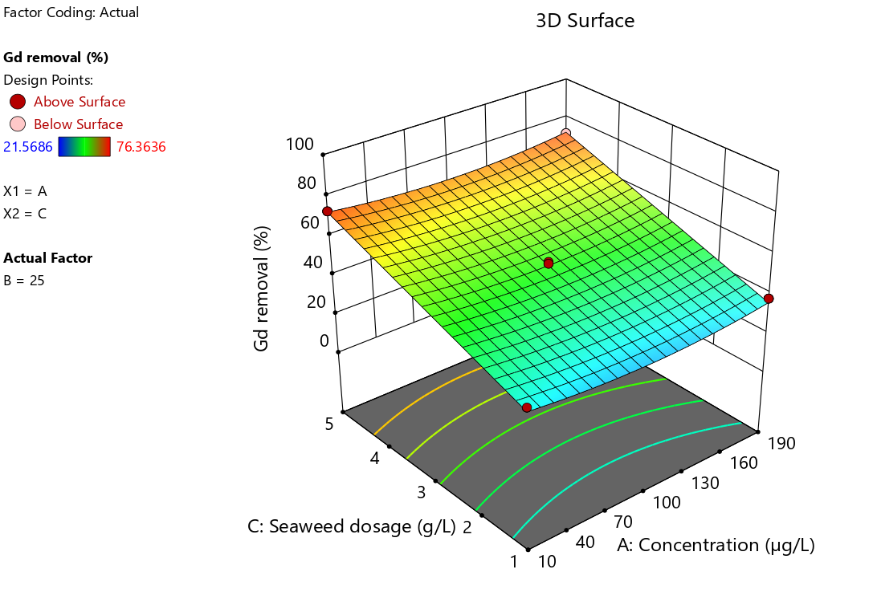


**Removal (%) of Ce at 24 h**

**C**

**B**

**A**

**Removal (%) of Gd at 24 h**

**E**

**D**

**F**


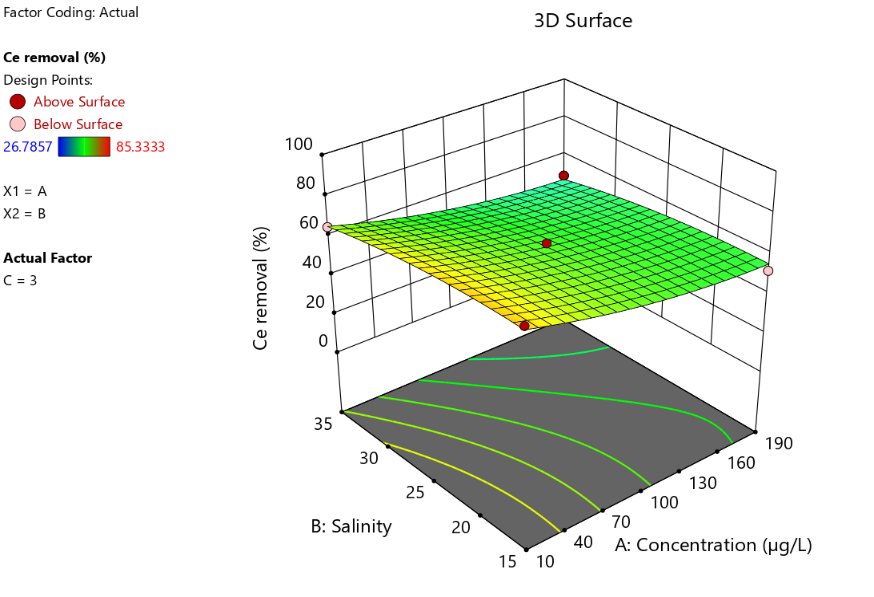

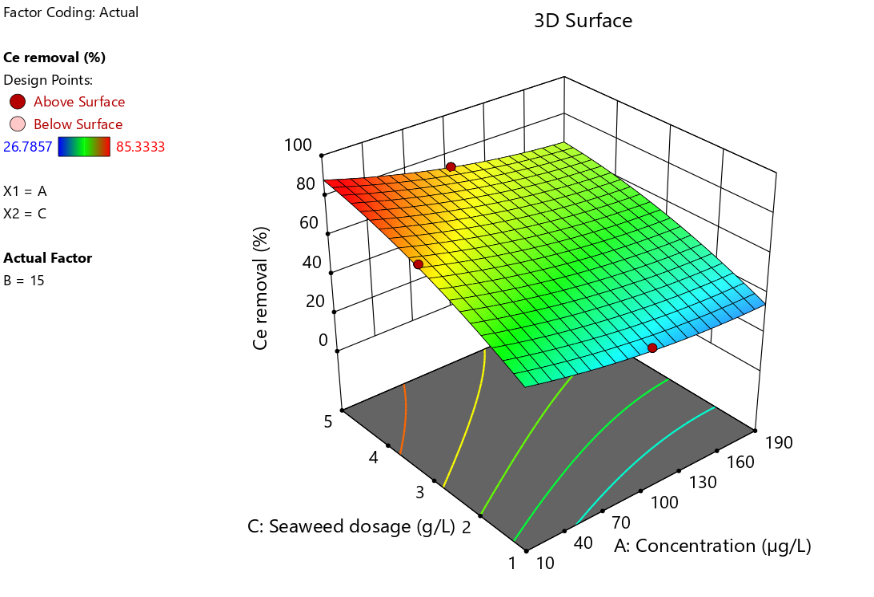

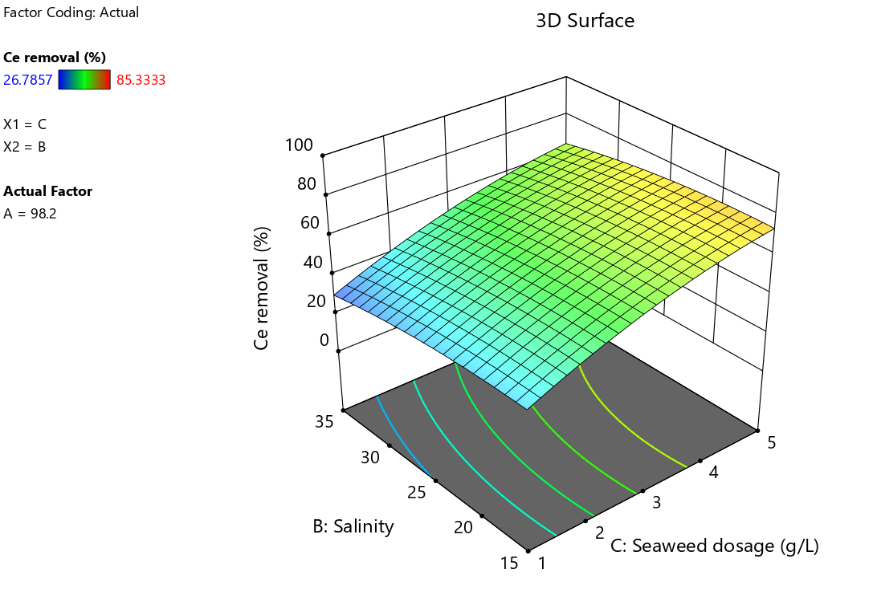

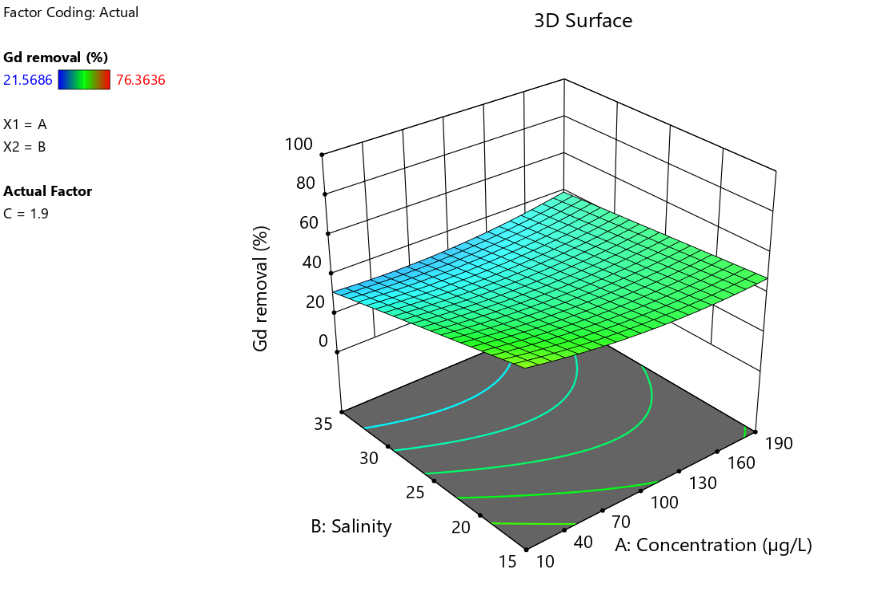

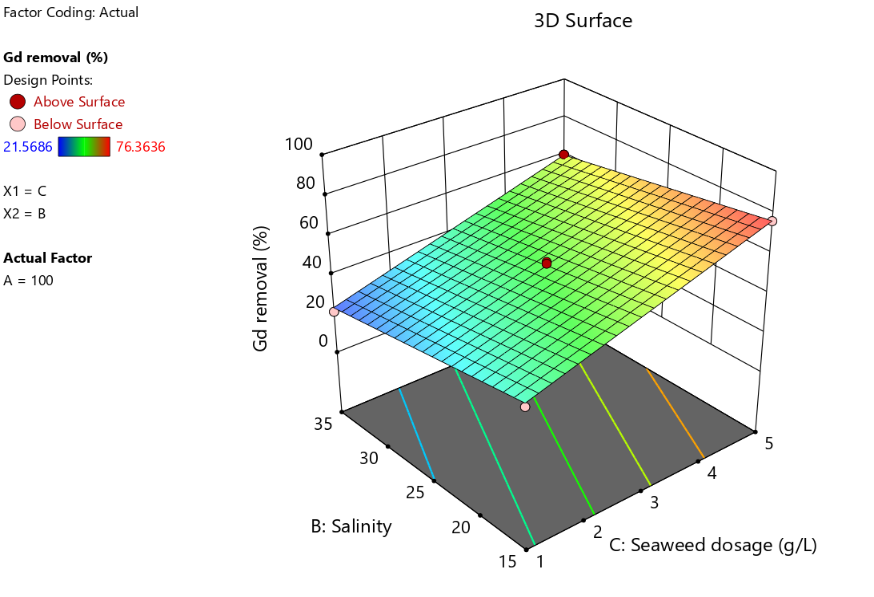


**F**

**C**

**Figure S2**: 3D response surface for the removal (%) of Ce and Gd by *Ulva* sp. at 24 h.

**Removal (%) of Eu at 24 h**


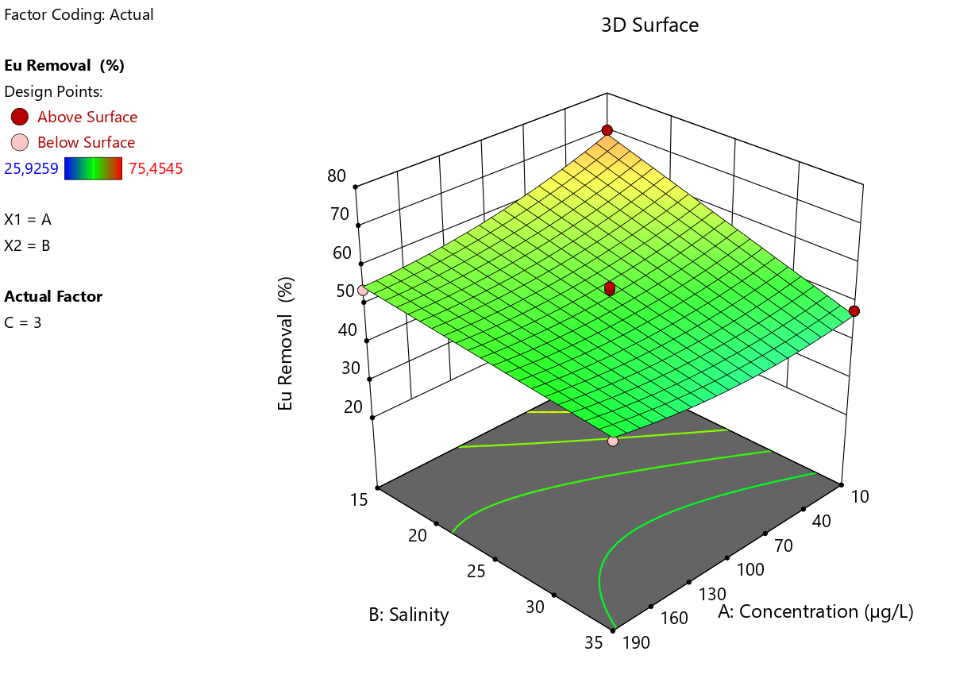


**A**


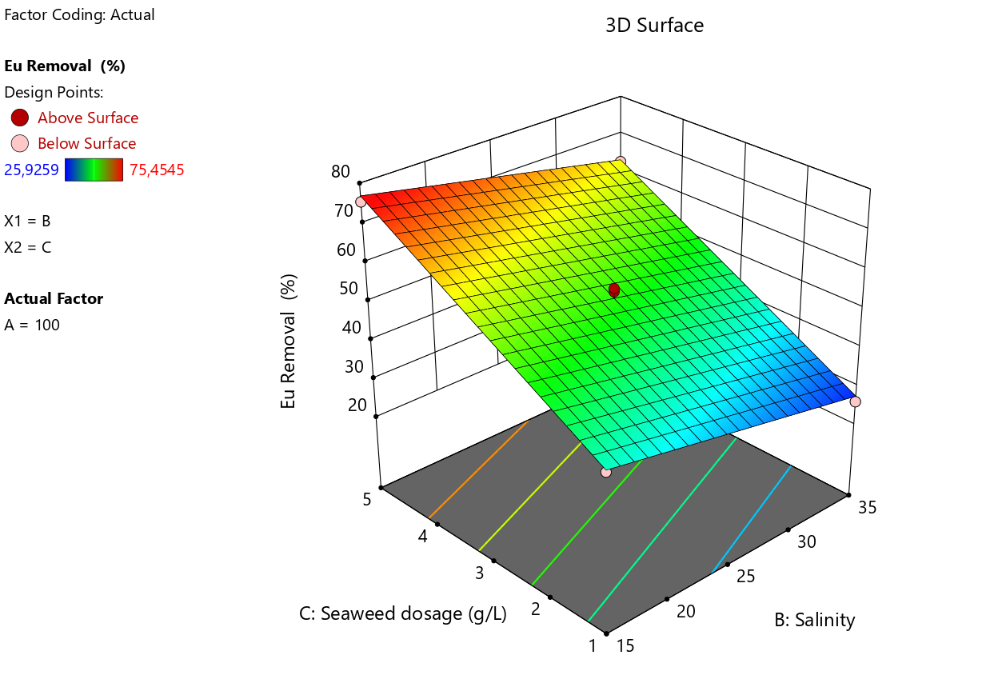


**C**


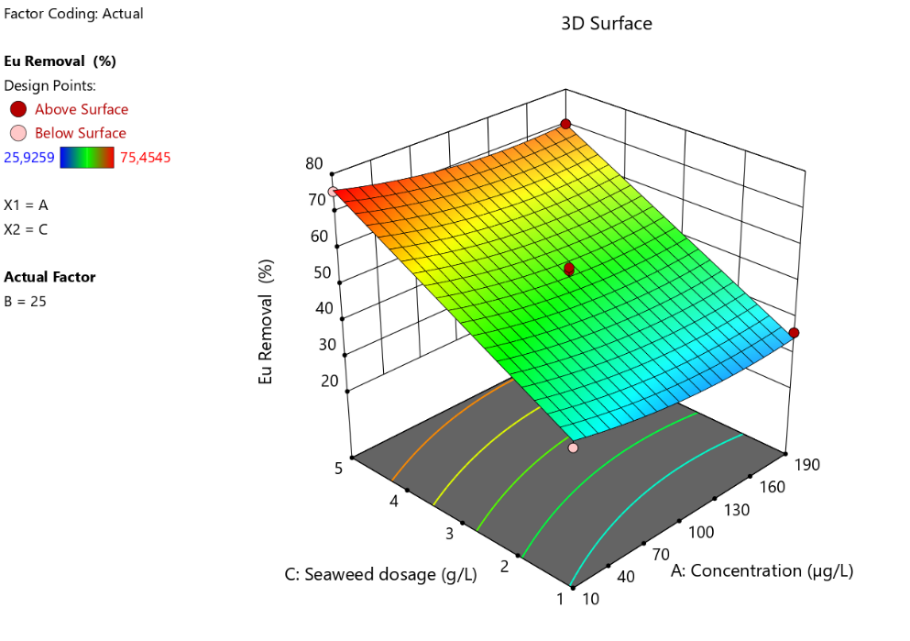


**B**

**Figure S3**: 3D response surface for the removal (%) of Eu and Tb by Ulva sp. at 24 h.

**Removal (%) of La at 96 h**

**C**

**B**

**A**


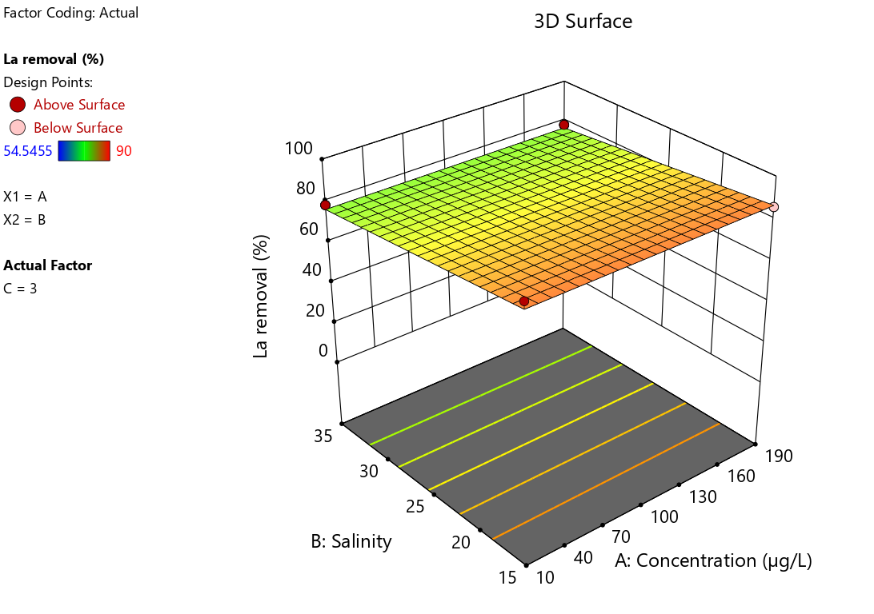

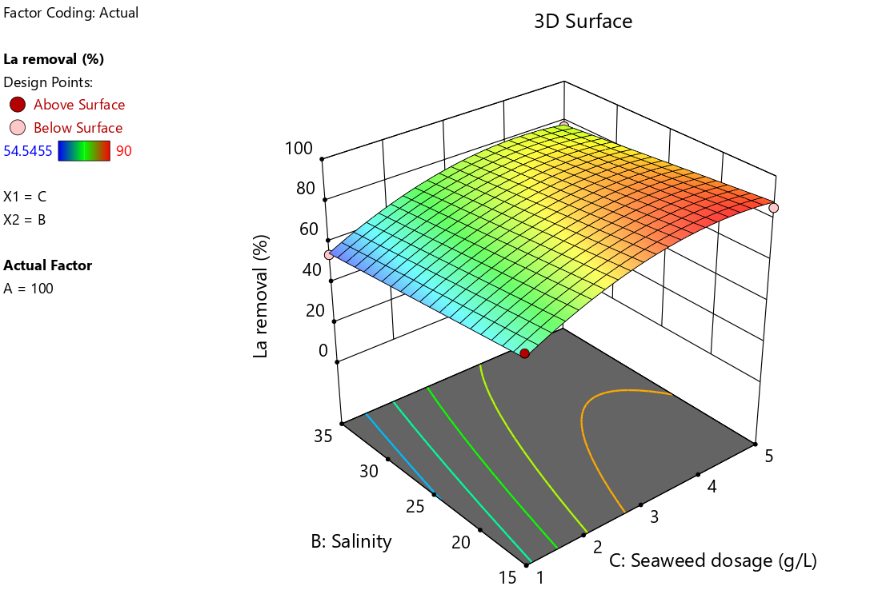

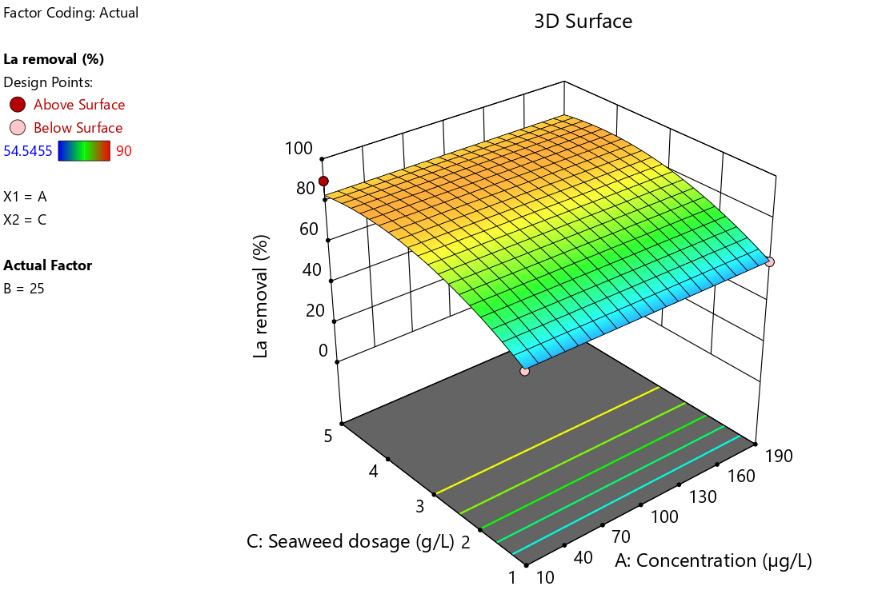


**Figure S4**: 3D response surface for the removal (%) of La by *Ulva* sp. at 96 h.

**Removal (%) of Gd at 96 h**

**E**

**D**

**F**

**Removal (%) of Ce at 96 h**

**C**

**B**

**A**


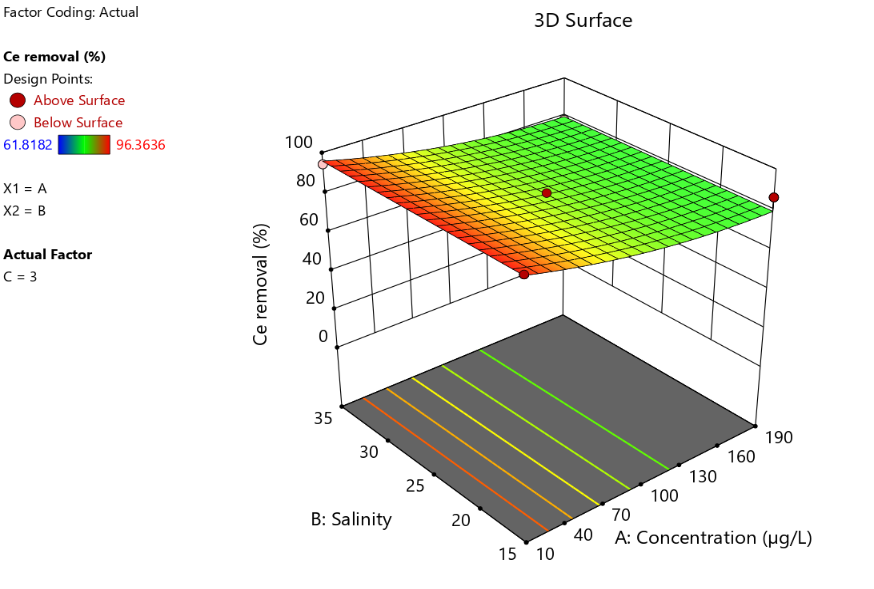

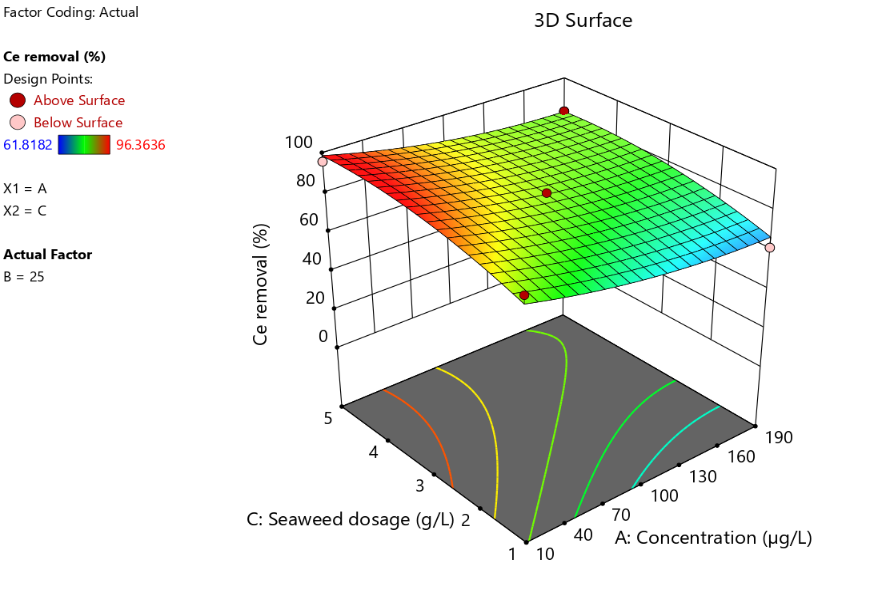

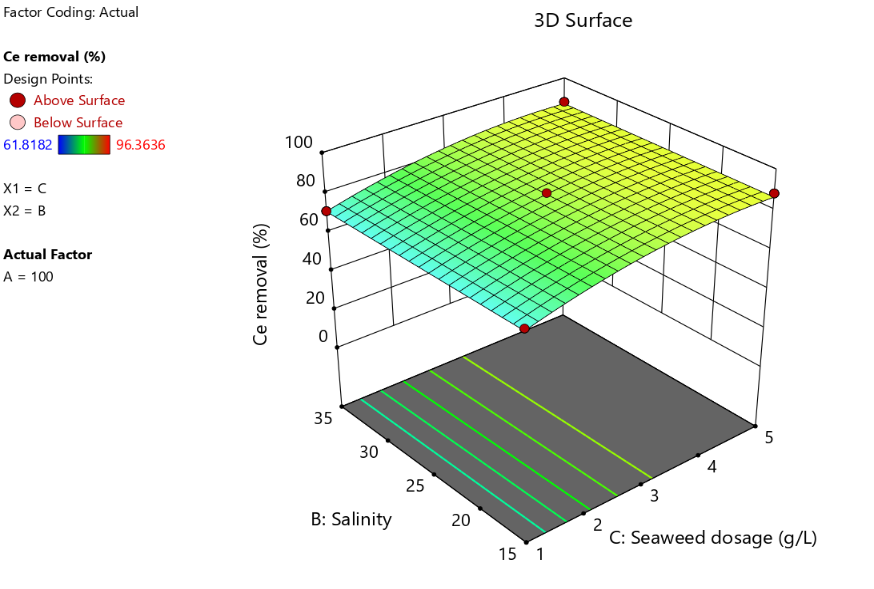

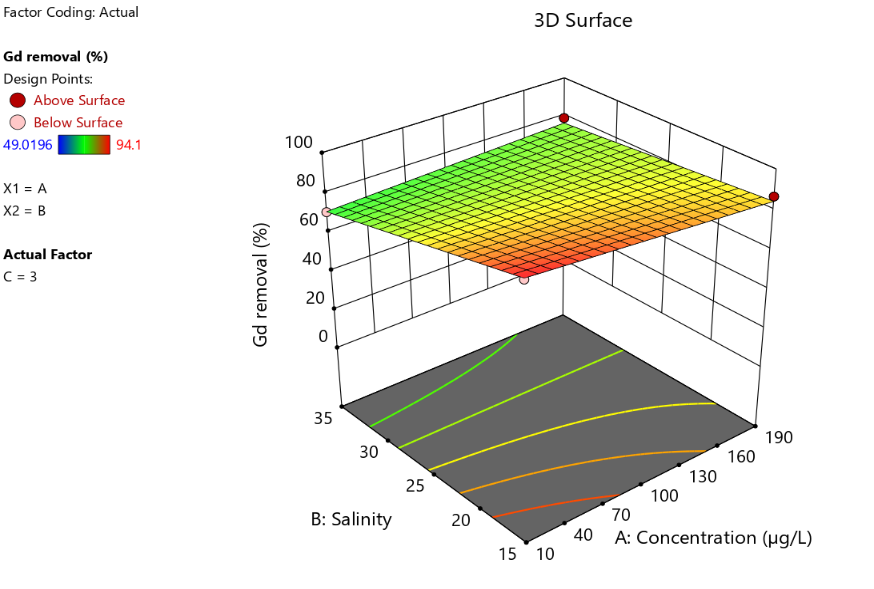

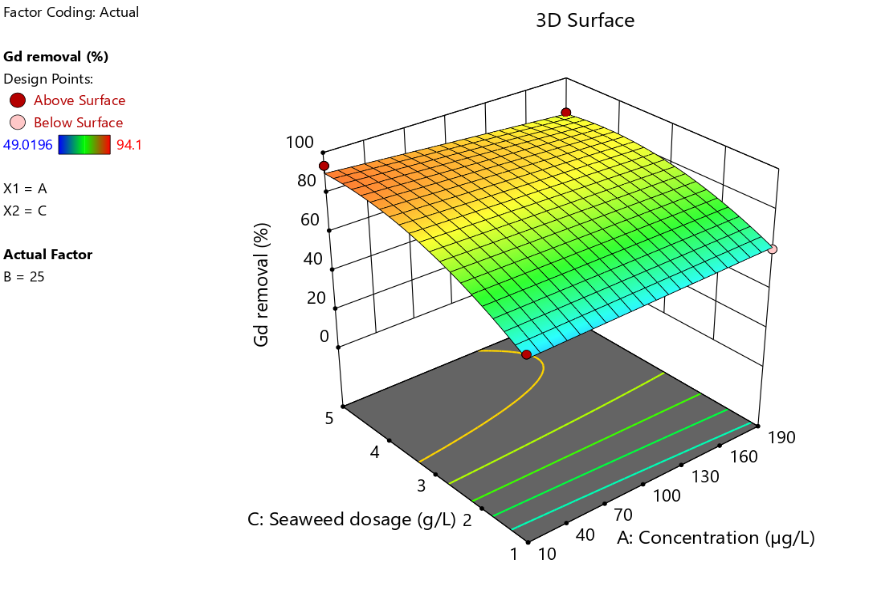

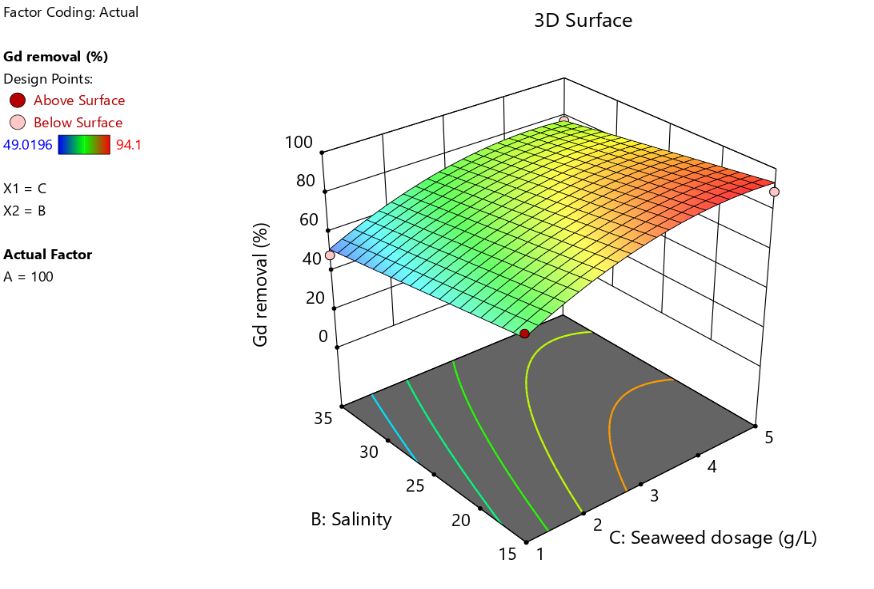


**A**

**Figure S5**: 3D response surface for the removal (%) of Ce and Gd by *Ulva* sp. at 96 h.

**Removal (%) of Eu at 96 h**


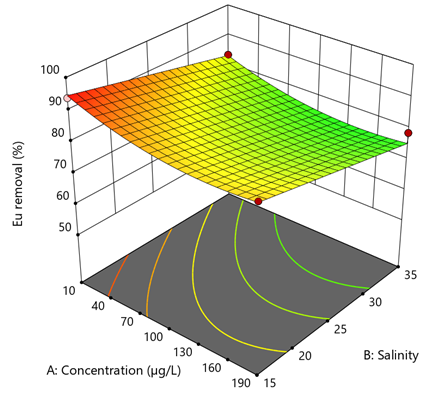


**A**


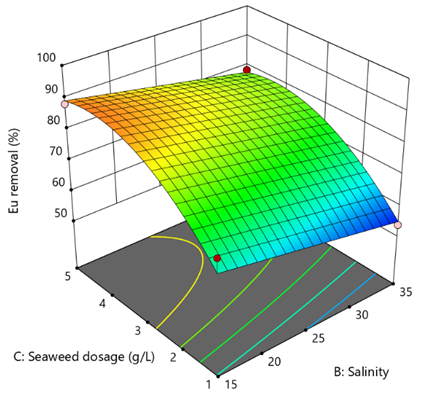


**C**


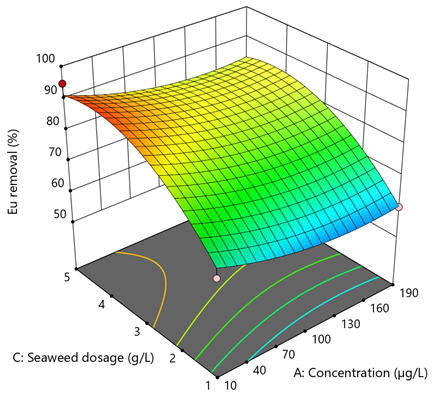


**B**

**Figure S6**: 3D response surface for the removal (%) of Eu and Tb by Ulva sp. at 96 h.

**Bioconcentration (µg/g) La at 24 h**

**E**

**D**

**F**

**Bioconcentration (µg/g) Y at 24 h**

**C**

**B**

**A**


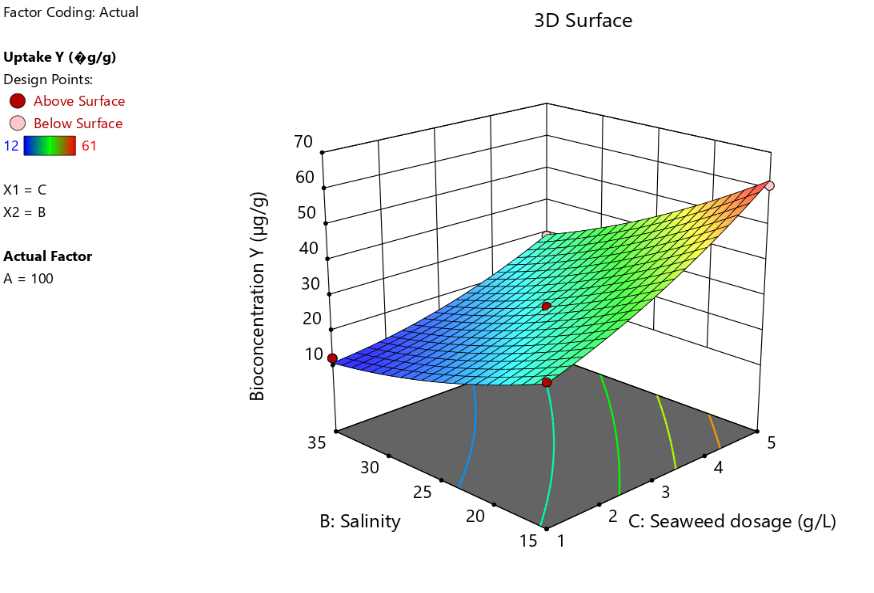

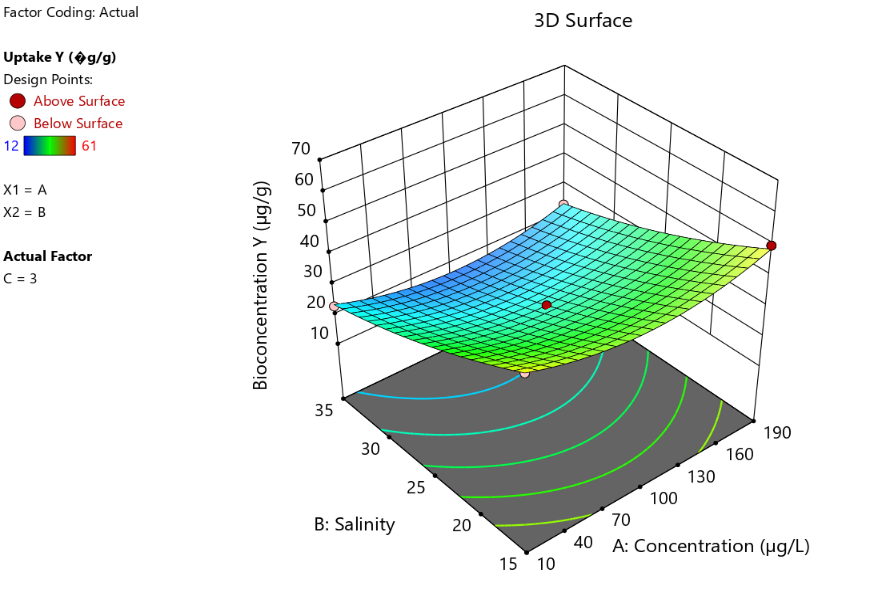

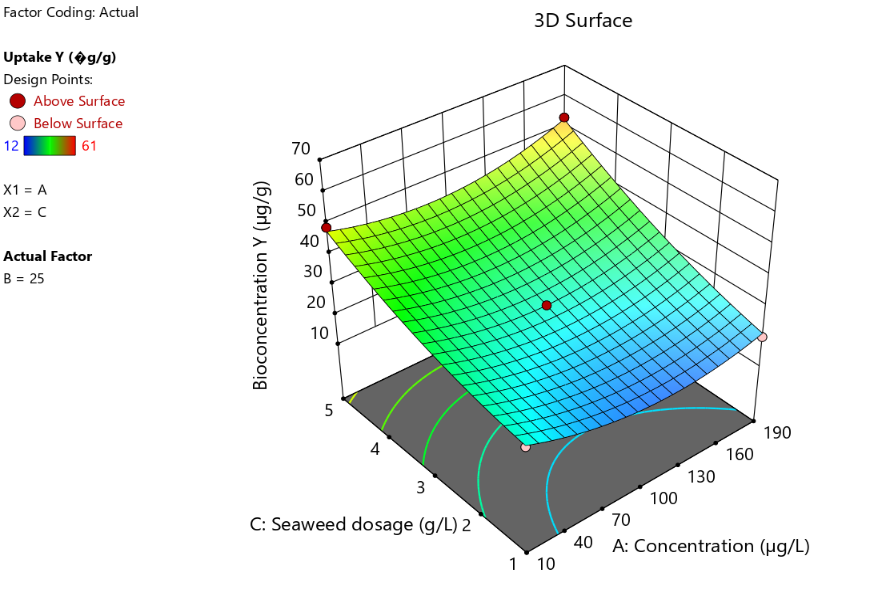

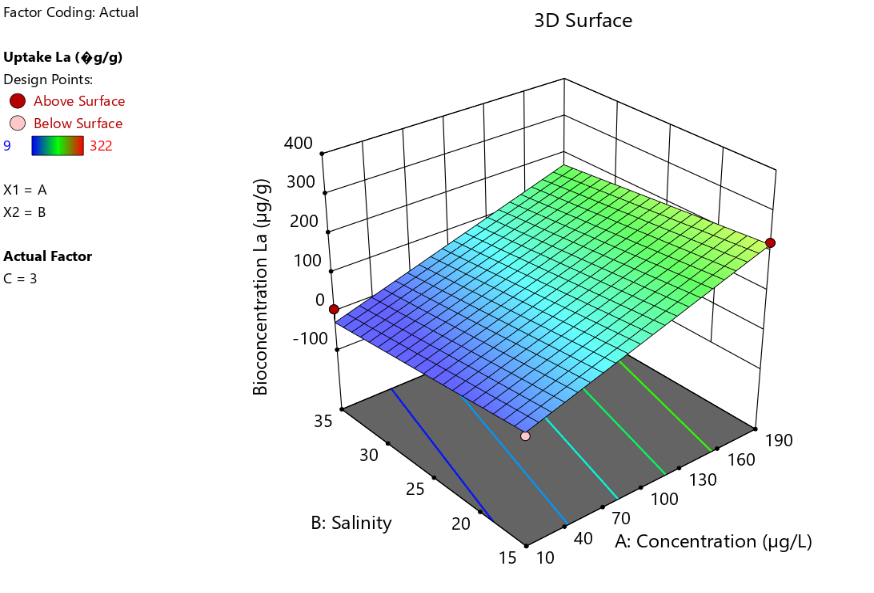

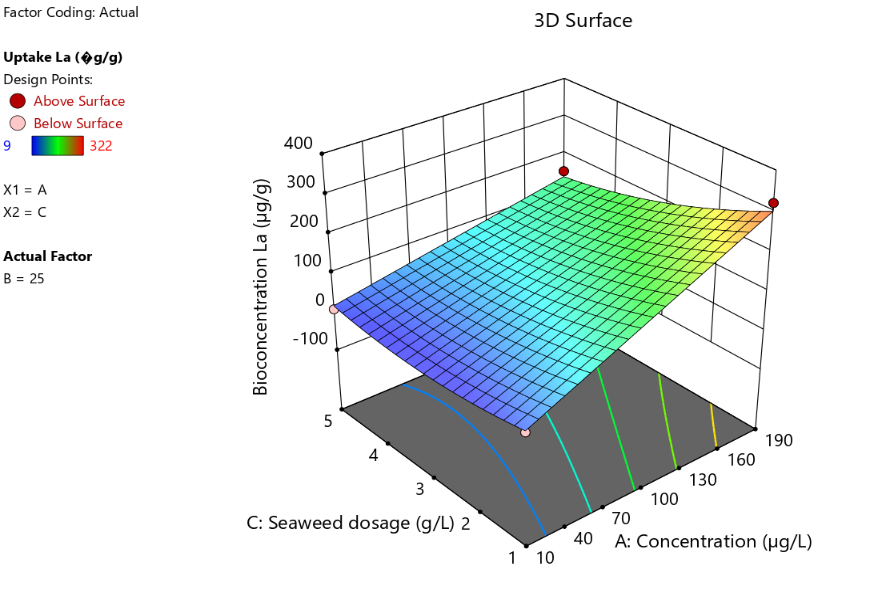

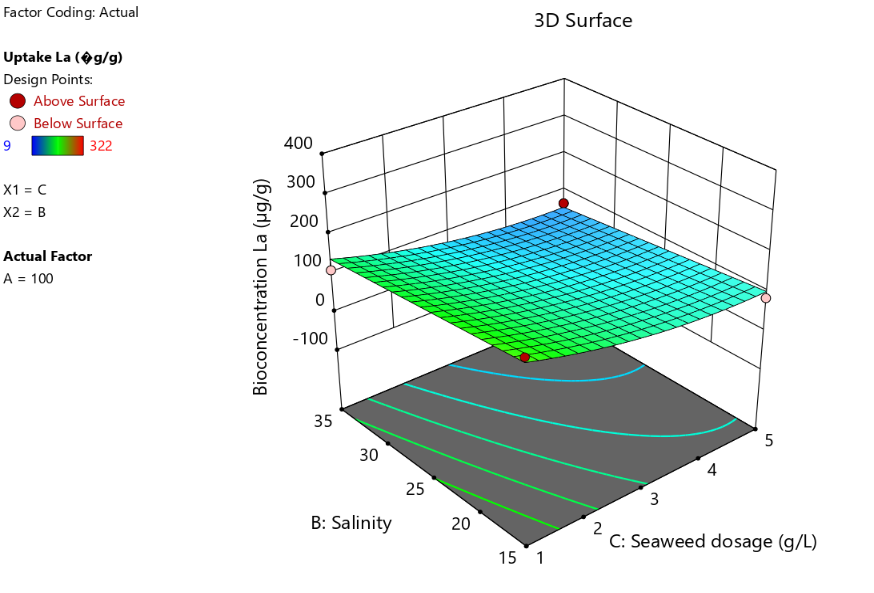


**Figure S7**: 3D response surface for the bioconcentration (q (µg/g)) of Y and La by *Ulva* sp. at 24 h.

**Bioconcentration (µg/g) of Ce at 24 h**


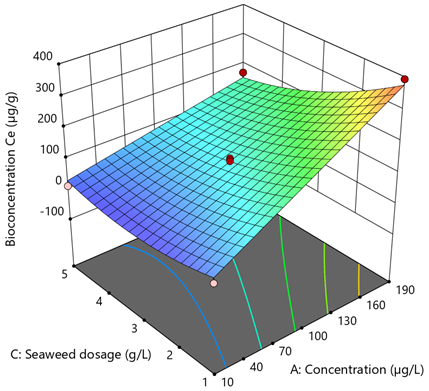


**A**


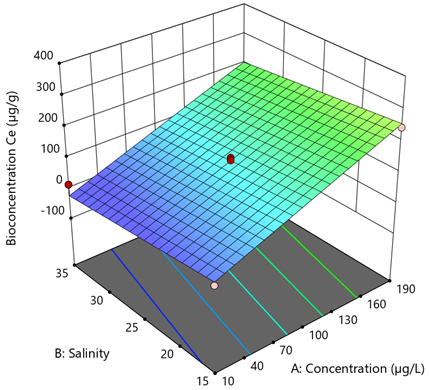


**C**


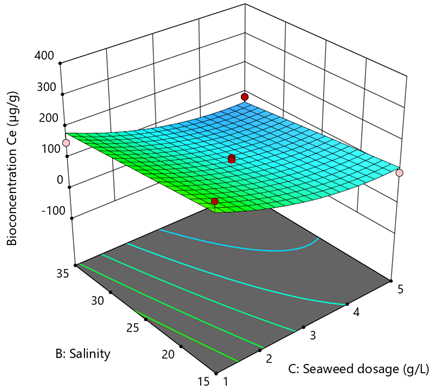


**B**


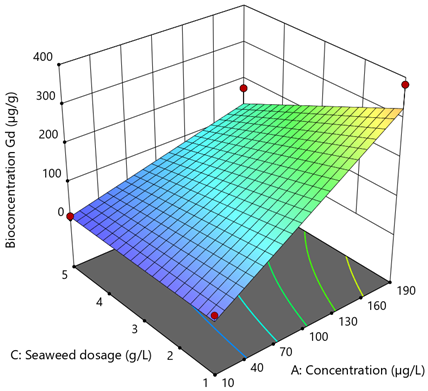


**D**

**Bioconcentration (µg/g) of Gd at 24 h**


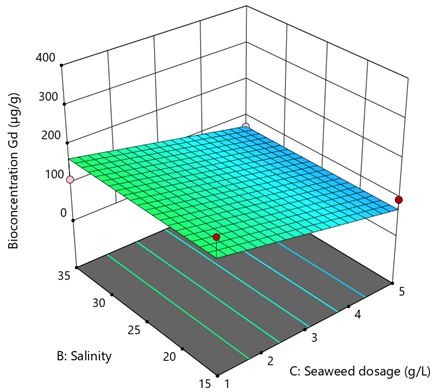


**E**


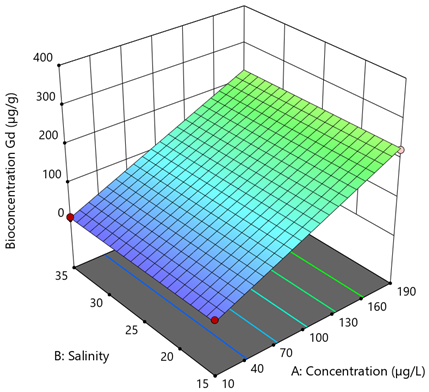


**F**

**F**

**Figure S8**: 3D response surface for the bioconcentration (q (µg/g)) of Ce and Gd by *Ulva* sp. at 24 h.

**Bioconcentration (µg/g) of Eu at 24 h**


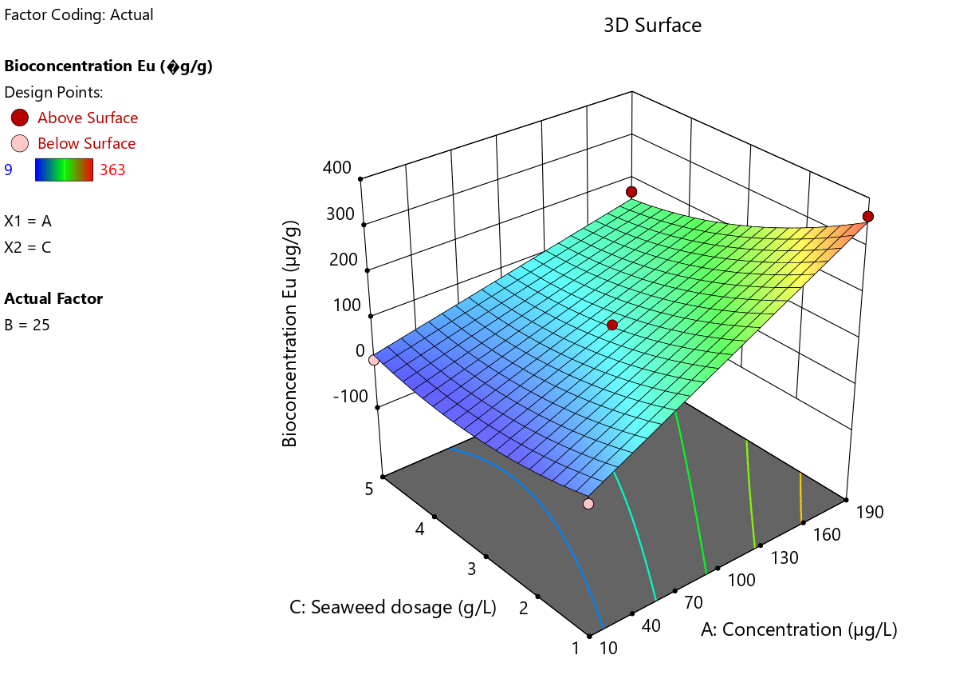


**A**


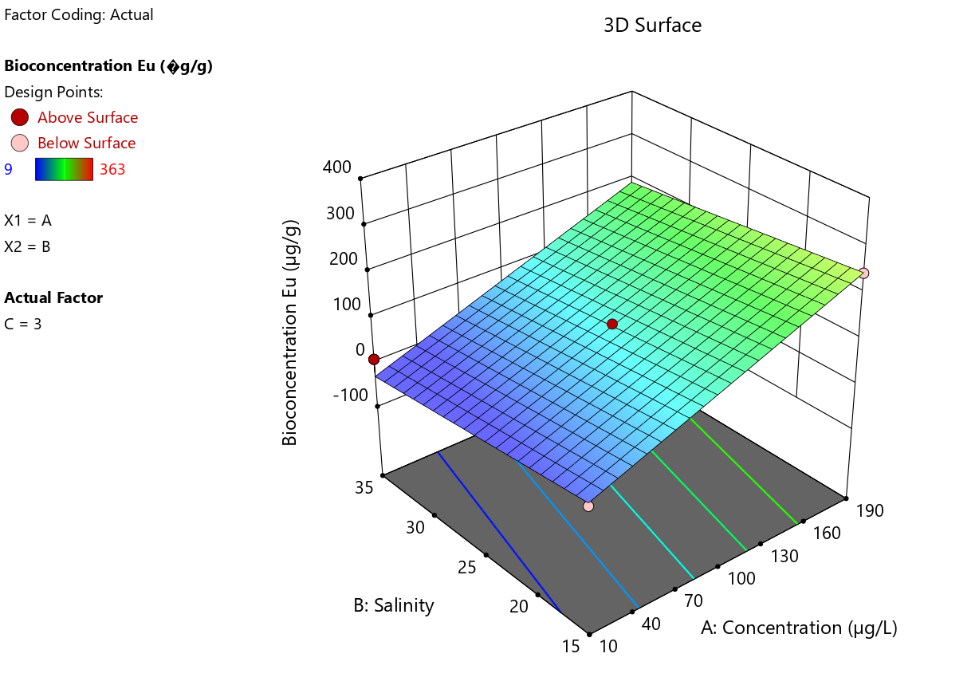


**C**


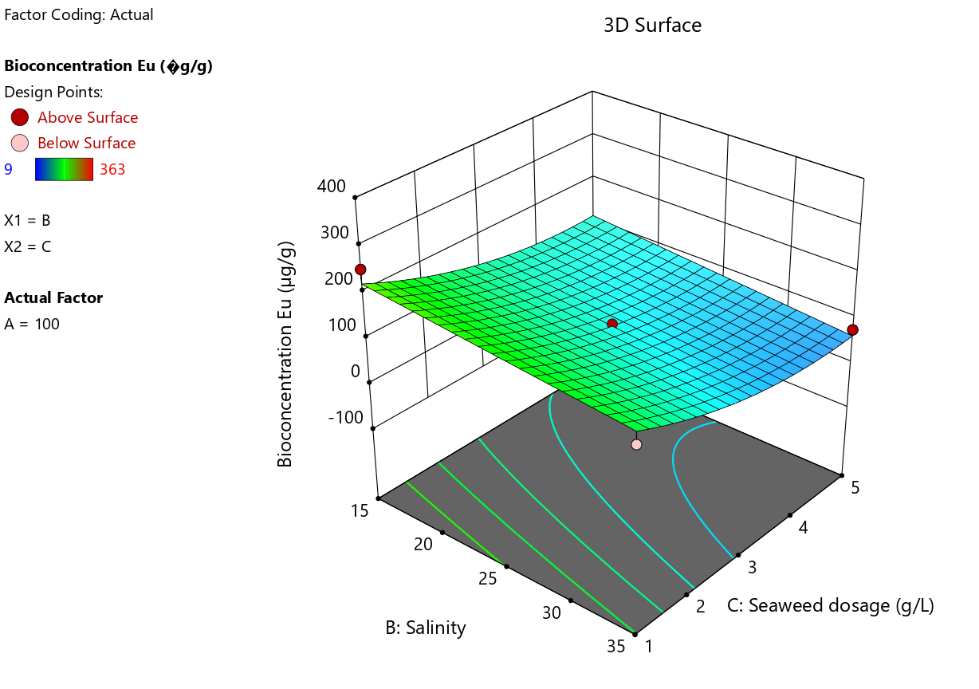


**B**

**Figure S9**: 3D response surface for the bioconcentration (q (µg/g)) of Eu and Tb by *Ulva* sp. at 24 h.

**Bioconcentration (µg/g) La at 96 h**

**C**

**B**

**A**


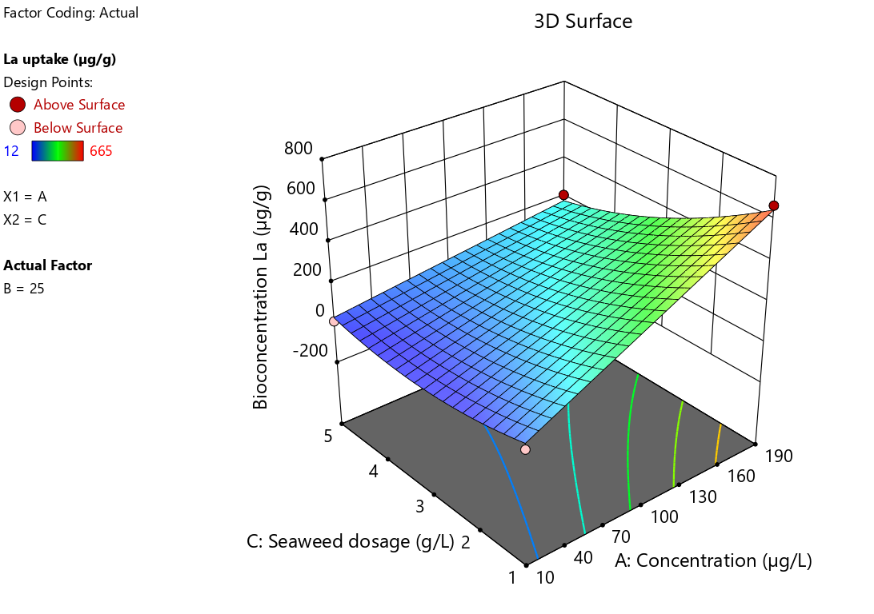

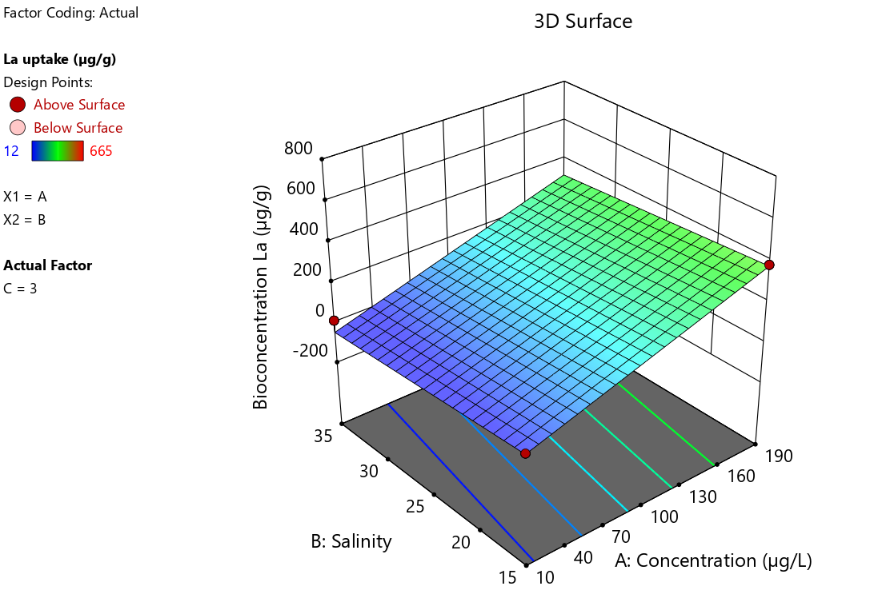

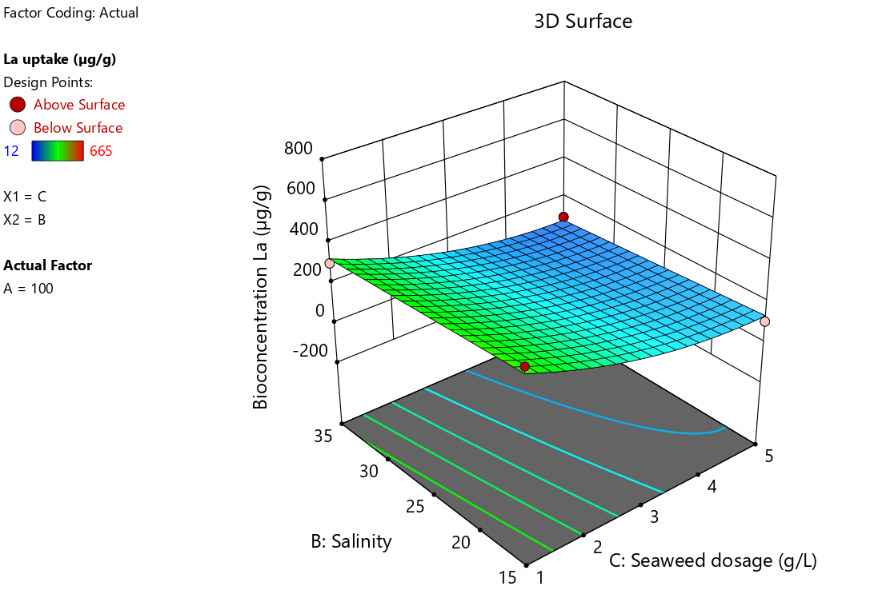


**Figure S10**: 3D response surface for the bioconcentration (q (µg/g)) of La by *Ulva* sp. at 96 h

**Bioconcentration (µg/g) of Ce at 96 h**


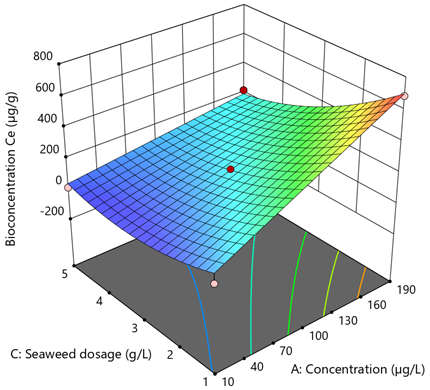


**A**


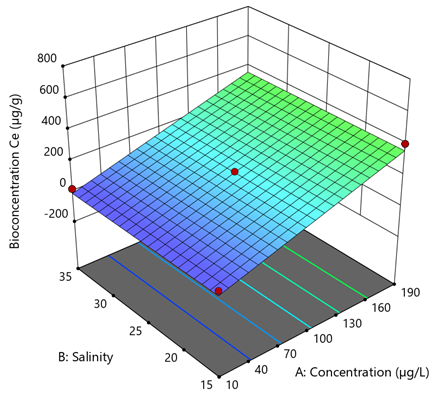


**C**


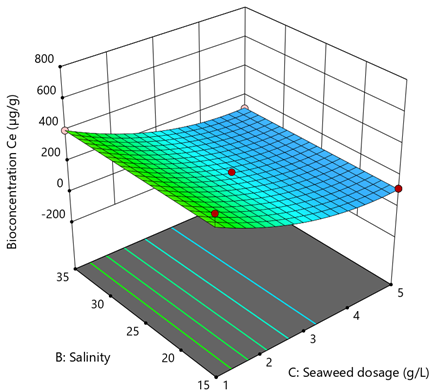


**B**


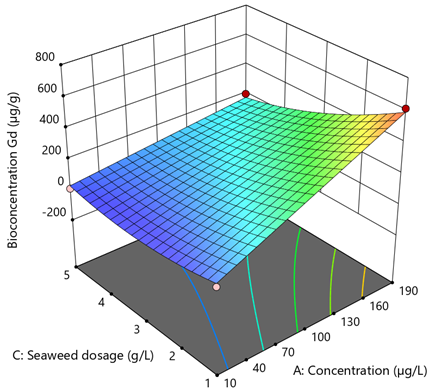


**D**

**Bioconcentration (µg/g) of Gd at 96 h**


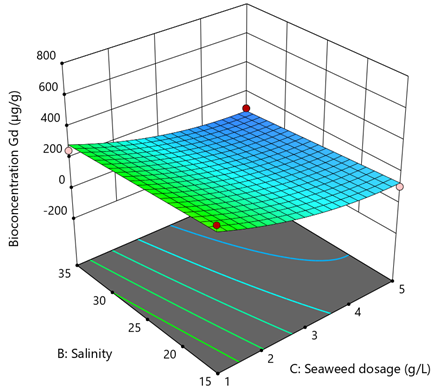


**E**


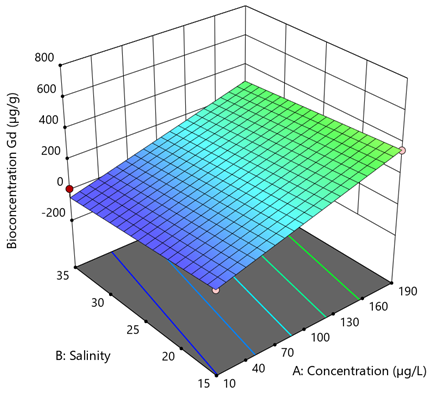


**F**

**Figure S11**: 3D response surface for the bioconcentration (q (µg/g)) of Ce and Gd by *Ulva* sp. at 96 h

**Bioconcentration (µg/g) of Eu at 96 h**


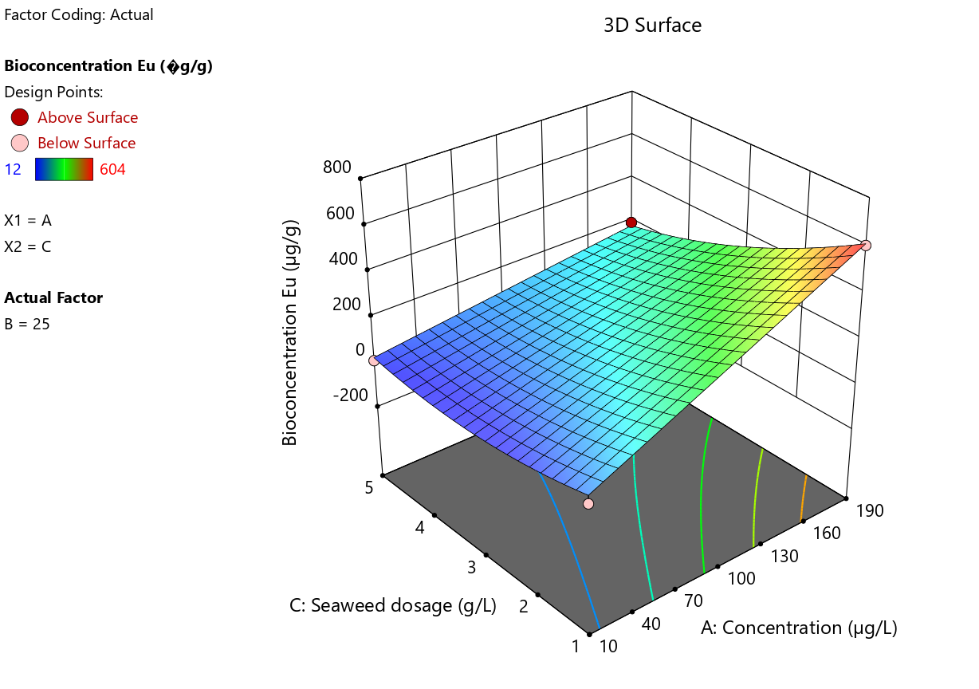


**A**


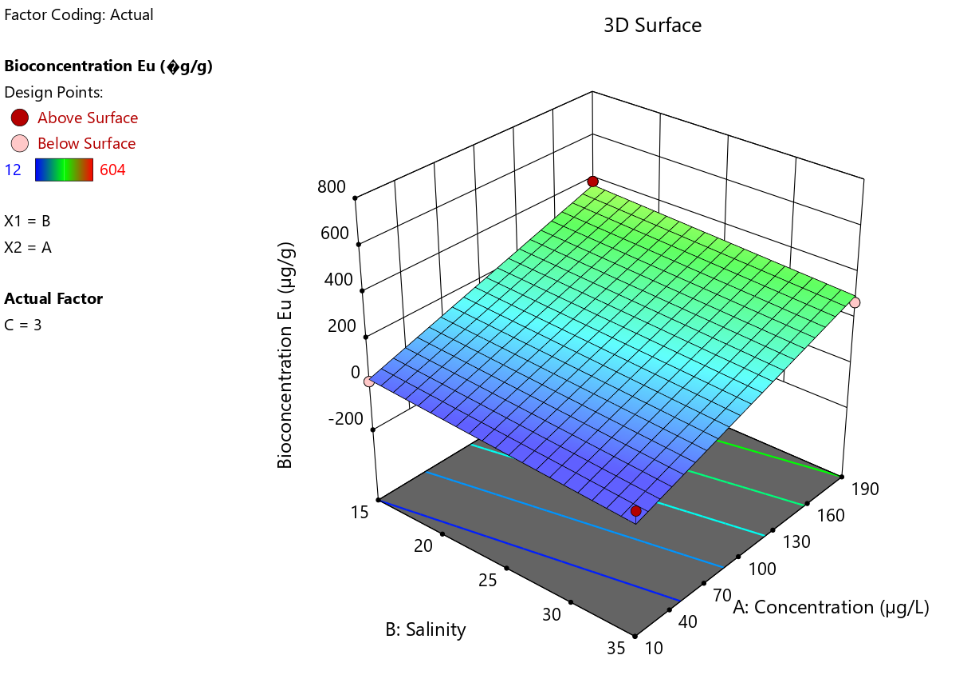


**C**


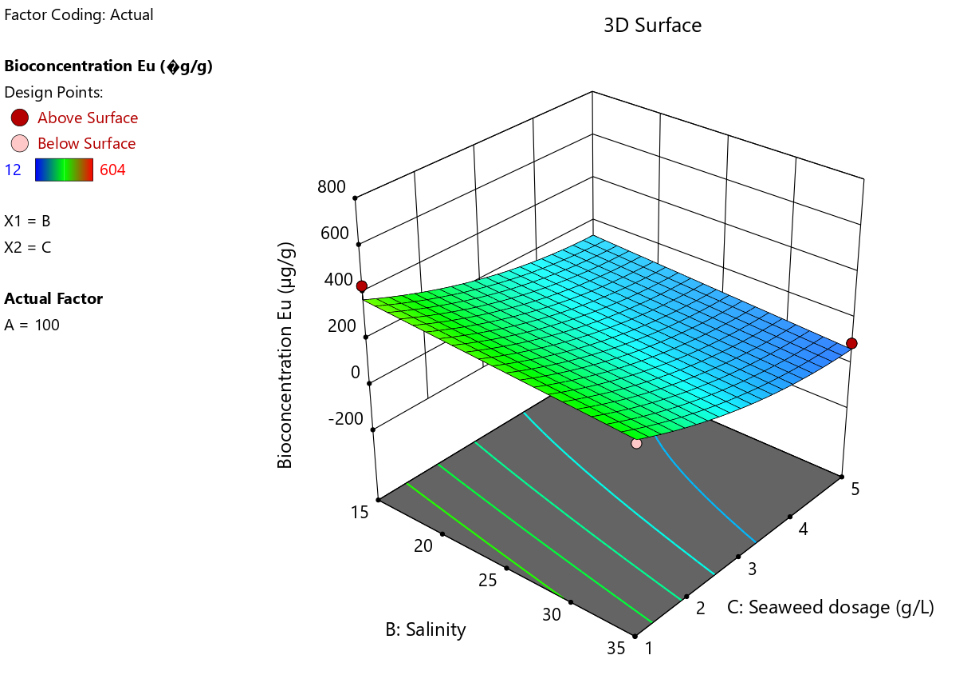


**B**

**Figure S12**: 3D response surface for the bioconcentration (q (µg/g)) of Eu and Tb by *Ulva* sp. at 96 h.
